# Harnessing gut microbiome enzymes: *Segatella copri* and *Stenotrophomonas maltophilia* prolyl peptidases degrade gliadin peptides and improve epithelial barrier function in a celiac disease model

**DOI:** 10.1128/spectrum.03214-25

**Published:** 2026-05-29

**Authors:** Bhagyashree Karmarkar, Dhiraj Dhotre

**Affiliations:** 1 National Centre for Cell Science, Savitribai Phule Pune University 29414, Pune, India; North Carolina State University, Raleigh, North Carolina, USA; Trevecca Nazarene University, Nashville, Tennessee, USA; Quaid-i-Azam University, Islamabad, Pakistan

**Keywords:** gut microbiome, celiac disease, prolyl peptidase, gliadin peptide, metagenome, rational selection

## Abstract

Celiac disease (CeD) is an autoimmune enteropathy triggered by gluten-derived peptides that resist gastrointestinal digestion, notably the proline-rich 33-mer and 11-mer gliadin epitopes. Here, we describe a rational, metagenome-based strategy to identify gut microbiome-derived prolyl peptidases capable of degrading these immunogenic peptides. Integrating metagenomic mining with structure-based *in silico* screening, we identified two novel enzymes PSP692 from *Segatella copri* and PSP464 from *Stenotrophomonas maltophilia*. Recombinant expression, purification, and characterization confirmed their activity under physiologically relevant conditions: PSP692 efficiently degrades the 33-mer at pH 6, while PSP464 targets the 11-mer at pH 4. Functional assays using CaCo-2 cell line, both in bi- and tri-dimensional assays, demonstrated that degradation of gliadin peptides by PSP692 and PSP464 significantly restored the expression of tight junction proteins (ZO-1 and occludin), reduced IL-6 secretion, and improved barrier integrity. These findings establish a foundational strategy for the discovery of microbiome-derived glutenases and provide both a compelling case and a methodology for data-driven discovery of functional enzymes that degrade immunogenic gliadin peptides, with translational potential as adjunct therapies in CeD and gluten-related disorders.

**IMPORTANCE:**

Celiac disease affects 1.4% of the global population, and, as of date, a gluten-free diet (GFD) is the only therapy available. Adherence to GFD is difficult, and inadvertent exposure to gluten still occurs. To address this, various approaches are utilized to develop adjuvant therapies. These include recombinant enzymes that, to date, have been discovered by serendipity. We have outlined and validated a method to identify enzymes with potential from metagenomic data, which will also be validated experimentally.

## INTRODUCTION

Celiac disease (CeD) is a chronic autoimmune disorder triggered by the ingestion of gluten, a storage protein found in wheat, barley, and rye (1). In genetically predisposed individuals (2–4), gluten-derived peptides, which are rich in proline and glutamine residues (5, 6), evade complete proteolysis in the gastrointestinal tract, leading to the activation of the adaptive immune system and subsequent duodenal inflammation. The only currently accepted treatment is a strict lifelong gluten-free diet (GFD), which is difficult to maintain and often fails to prevent inadvertent gluten exposure. Consequently, there is a pressing need for adjunctive therapeutic strategies that can mitigate gluten toxicity *in situ*, particularly by degrading immunogenic gluten peptides prior to their interaction with the mucosal immune system.

The human gut microbiome plays a pivotal role in both the exacerbation and alleviation of CeD pathogenesis (7). Dysbiosis, characterized by the expansion of pro-inflammatory taxa and loss of beneficial commensals, can potentiate disease activity by enhancing intestinal permeability and generating immunogenic gluten-derived peptides through unregulated proteolysis (8). Conversely, commensal bacteria, such as *Bacteroides*, *Prevotella*, and *Lactobacillus* spp., may harbor intrinsic prolyl-specific peptidases that contribute to gluten detoxification by cleaving proline- and glutamine-rich motifs into less immunogenic fragments (9). Several studies now support that the functional potential of the microbiome, rather than its taxonomic composition alone, critically shapes host responses to dietary gluten and influences disease outcomes (10). Leveraging this microbial enzymatic repertoire through metagenomic mining and structure-guided discovery has already uncovered novel peptidases. Recent synthetic biology approaches extend this concept further, demonstrating that genetically engineered commensal gut bacteria can be tailored to stably express certain enzymes in situ (11), thereby providing sustained protection against inadvertent gluten exposure, while other approaches use recombinant enzymes. Together, these insights highlight the gut microbiome as both a key contributor to CeD pathogenesis and a promising reservoir of therapeutic enzymes.

Several microbial enzymes, particularly prolyl endopeptidases (PEPs), dipeptidyl peptidase IV (DPPIV), and other serine proteases (12–14), have been investigated for their ability to cleave the immunodominant 33-mer gliadin peptide (LQLQPFPQPQLPYPQPQLPYPQPQLPYPQPQPF). However, most of the current research remains limited to the characterization of enzymes discovered through labor-intensive functional screening, which inherently constrains the pool of candidates advancing toward clinical application. In contrast, our study advocates for a more rational and data-centric approach by integrating metagenomic data with *in silico* predictive tools to pre-select promising glutenases based on structural and functional criteria.

In this study, we leveraged metagenomic data and peptidase databases to identify and characterize microbial proteases with gluten-degrading potential. Candidate enzymes (PSP692 and PSP464) were selected based on their structural similarity to known glutenases and predicted activity against gliadin epitopes, as determined through docking and molecular dynamics simulation. These two promising enzyme candidates were cloned and expressed recombinantly, followed by purification and biochemical characterization. Enzymatic assays confirmed their activity against synthetic gliadin peptides. Furthermore, immunological assays using the Caco-2 cell line demonstrated a reduction in gliadin-induced inflammatory markers, indicating that the enzymatic cleavage products were rendered non-immunogenic. Notably, we evaluated enzyme activity against both full-length and truncated gliadin immunogenic peptides, including the 33-mer and 11-mer epitopes (15). Our findings demonstrate that PSP692 exhibits robust activity against the full-length 33-mer, while PSP464 preferentially degrades the 11-mer fragment (GPQQSFPEQEA), highlighting their complementary substrate specificities. This dual-targeting strategy represents a departure from conventional single-enzyme approaches that target only the 33-mer and support the development of enzyme cocktails tailored for comprehensive gluten detoxification.

Taken together, our study has validated two enzyme candidates derived from metagenomic data and peptidase databases, expanding the repertoire of microbial glutenases and demonstrating the utility of our data-centric approach over the conventional activity screens. These findings provide a foundational step toward the rational selection and development of complementary enzyme supplements that can degrade dietary gluten *in vivo*, thereby reducing the immunogenic burden in CeD patients.

## MATERIALS AND METHODS

### Data pre-processing

**Fig 1:**
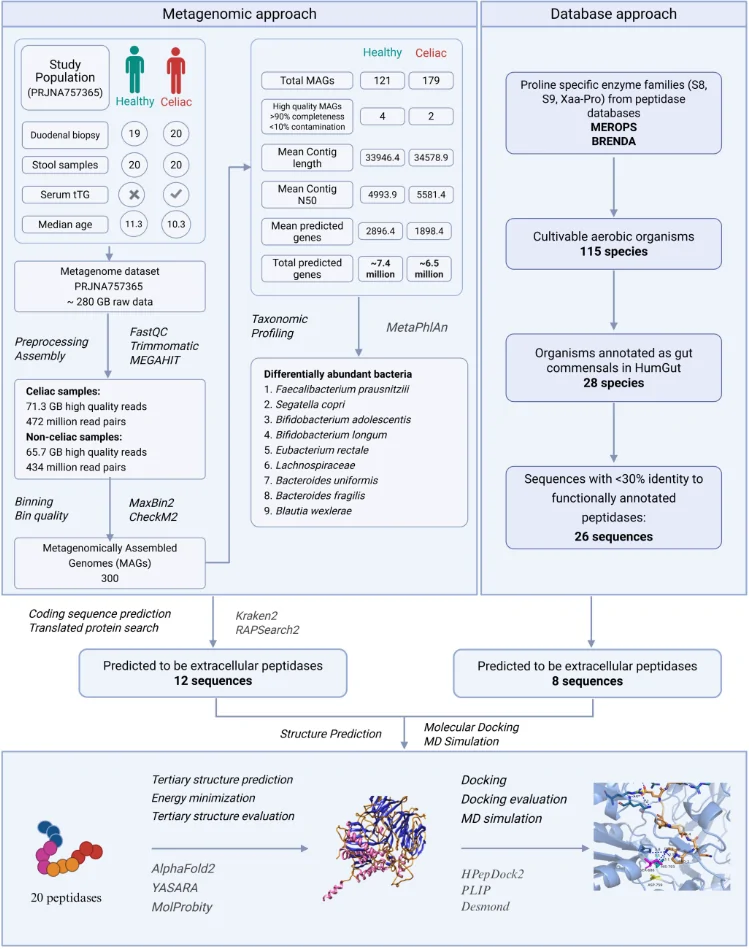
Reads of celiac metagenome data set PRJNA757365 (16, 17) were checked for quality using FastQC, and assembly was done using MEGAHIT (20). The contigs were sorted into bins using MaxBin2 (21), which identified 121 bins, i.e., organisms present in healthy control samples, and 179 such bins in samples from celiac patients. Contigs likely containing coding sequences were identified by Kraken2 (24), and the presence of an open reading frame (ORF) was determined by the eggNOG-mapper module of HumanN3.6 (24). RAPSearch2 (25) was used to perform a translated alignment, and from the result file, sequences annotated as belonging to proline-specific peptidase enzyme families and present in differentially abundant organisms were selected. PSPs predicted to be extracellular, and thus more likely to have a role in degradation of gut luminal gluten/gliadin, by a consensus of three tools—SignalP6 (26), PrediSi (27), and Phobius (28)—were submitted for tertiary structure prediction (ST2C). From the MEROPS (29) and BRENDA databases, 26 PSPs present in cultivable organisms and annotated to be non-pathogenic gut commensals were used for *in silico* analysis. Twelve peptidase sequences from metagenome and eight from databases underwent *in silico* screening to identify high potential candidates for experimental validation.

Data submitted under NCBI BioProject ID PRJNA757365 (16, 17) and PRJNA486782 (18) were downloaded and analysed with in-house methodology for taxonomic abundance profiling, quality assessment, assembly and binning, gene classification, and translated protein search. Briefly, read files of studies on CeD stool metagenomes, with data sets PRJNA757365 and PRJNA486782 as the only publicly available ones, were downloaded. The quality was assessed using FastQC (http://www.bioinformatics.babraham.ac.uk/projects/fastqc/), and it was determined that no read trimming is required. Taxonomic abundance was determined using MetaPhlAn2 (19), assembly was performed with MEGAHIT (20), and binning was conducted using MaxBin2 (21). Bins identified as belonging to organisms in higher abundance in non-CeD individuals by CheckM (22) were submitted to Kraken2 (23) for gene classification. Contigs containing coding regions were identified in the “classified” output of Kraken2, and the presence of ORFs was determined using the eggNOG-mapper module of HumanN3.6 (24). The RAPSearch2 (25) module was then utilized for translated protein similarity search using the UniRef90 database. Proteins from the output that fit our in-house criteria (Fig. 1) were selected for *in silico* evaluation.

### Database analysis

To identify candidate gluten- and gliadin-degrading enzymes with no prior annotation for such activity, we analysed data from two databases: BRENDA (30) (the Comprehensive Enzyme Information System) (https://www.brenda-enzymes.org/) and MEROPS (the Peptidase Database) (https://www.ebi.ac.uk/merops/) (29). BRENDA was used to screen for hydrolases, specifically peptidases, based on their EC numbers, substrate specificity, and reported kinetic parameters. Enzymes with biochemical properties suggestive of activity against proline- and glutamine-rich substrates were shortlisted. In parallel, MEROPS was used to analyze peptidases at the family and clan levels, with a focus on serine peptidases previously implicated in gliadin cleavage. MEROPS data on active site residues, substrate cleavage sites, and known inhibitors were used to further refine enzyme selection.

For all shortlisted enzymes, the source organism was identified and cross-checked against literature and publicly available microbiome data sets to confirm whether it is a cultivable, non-pathogenic commensal of the human gut. Only enzymes meeting these criteria were retained for downstream structural, *in silico* analysis, and experimental validation. This multi-tiered selection strategy enabled the identification of functionally probable, previously uncharacterized gluten-degrading enzymes from gut-relevant microbial sources.

### *In silico* evaluation

Sequences of peptidases obtained from databases and metagenome data analysis were submitted to AlphaFold2 (31) for tertiary structure prediction. All the models were subjected to energy minimization using SwissPDBviewer (32) and evaluated using MolProbity (33). The top-ranked model of each peptidase was used for docking. Docking of the predicted tertiary structure of the peptidase was performed using the HPepDock (34) server. The ligands were selected from PDB based on the following criteria: peptide ligand must be bound to either MHC or TCR, and structure must be determined by X-ray crystallography with a resolution <2 Å. The docked structures were evaluated using PLIP (35) (Protein Ligand Interaction Profiler), and peptidases whose catalytic residues formed a donor hydrogen bond of <3 Å with the ligand were selected for MD simulation. Prediction of active site residues was performed using GASS (36) (http://gass.unifei.edu.br). After tertiary structure prediction and docking, structures in which the enzyme catalytic residues were predicted to form a donor hydrogen bond of length less than 3 Å were selected for MD simulation. MD simulation was performed with Desmond (37) academic version 2021-1 via Schrodinger Maestro GUI.

### Cloning of prolyl peptidase genes

Genes encoding for PSP464 and PSP692 were amplified from the genomic DNA of *Stenotrophomonas maltophilia* MCC2084 and *Prevotella copri* DSM18205, respectively. Primer sequences used for amplification were as follows: (i) PSP464-F 5’- ATTGGATCCATGCTCATCCGTCGAAC-3′; (ii) PSP464-R 5’- CTAAAGCTTCTTGGCGTTCTTCACGTAG-3′; (iii) PSP692-F 5′-CGGGATCCGATGAAAAGATTTCCAGTTTTTATTGC-3′; and (iv) PSP692-R 5’- CCCAAGCTTCTTCAGATTCTGCTTGAACCAGTTA-3′. The amplified genes were cloned into a pET22b plasmid (Novagen Healthcare Pvt. Ltd.).

### Expression and purification of prolyl peptidases

Recombinant expression plasmids were introduced into *Escherichia coli* BL21(DE3) cells via transformation. Transformants were grown at 37°C and induced in the presence of 0.5/1 mM IPTG at 18°C overnight. Lysis was performed by pelleting down induced cultures and resuspending in SDS (final concentration 1% [wt/vol]) solution, followed by centrifugation to collect supernatant. Equal amounts of protein from uninduced and induced cell lysate were analysed on a 12% SDS-PAGE gel, transferred to a nitrocellulose membrane, and probed with anti-His antibody (Cat no: 2366T, CST Inc.) to confirm expression of full-length recombinant protein.

All purification steps were performed at 4°C unless noted otherwise. Recombinant bacterial culture was pelleted down at 4,000–5,000 × *g*, supernatant was discarded, and cells were lysed by resuspension in a solution with a final concentration of 1% (wt/vol) SDS. The lysate was centrifuged at ~12,000 × *g* for 30 min, and the supernatant was collected for purification. The volume of Ni-NTA magnetic beads (GenScript) added to the suspension was one-third of the total volume of lysate; the mixture was then spun on rotospin at 15 rpm for at least 1 h. Elution was performed using reaction buffers of recombinant enzymes that did not contain any imidazole.

### Evaluation of purified enzyme activity

Post-proline cleavage activity of the purified enzymes was assessed using two chromogenic substrates: Z-Gly-Pro-pNA (Sigma-Aldrich Chemicals Pvt. Ltd.) and a synthetic peptide substrate Z-PPF-pNA, which was custom-synthesized at >95% HPLC purity (Genetoprotein Pvt. Ltd.). Z-Gly-Pro-pNA was prepared as a 50 mM stock solution in dimethyl sulfoxide (DMSO) and stored at –20°C until use. Z-Pro-Pro-Phe-pNA was prepared as a 20 mM stock solution in nuclease-free water and stored at −20°C until use. Hydrolytic activity against Z-PPF-pNA was evaluated for enzyme PSP464 in 100 mM glycine-HCl buffer at pH 4.0, while Z-Gly-Pro-pNA hydrolysis by enzyme PSP692 was measured in 1× phosphate-buffered saline (PBS) at pH 6.0. Enzymatic cleavage of the pNA-linked substrates was monitored spectrophotometrically by measuring the increase in absorbance at 410 nm, which corresponds to the release of p-nitroaniline upon substrate hydrolysis.

Each reaction was carried out in a final volume of 100 µL in thin-walled 0.5 mL PCR tubes and contained a final enzyme concentration of 1 µM. Substrate and enzyme solutions were added, such that their combined volume did not exceed 50% of the total reaction volume. The concentration of DMSO was carefully maintained below 0.5% in all assays to minimize solvent effects on enzyme activity. Absorbance values obtained were used to calculate enzymatic activity based on a standard curve generated with known concentrations of p-nitroaniline in the respective assay buffers. Background absorbance from substrate auto-hydrolysis was accounted for by subtracting the optical density (OD_410_) of control wells containing substrate without enzyme. All measurements were performed in triplicate to ensure reproducibility.

### LC-MS analysis of gliadin peptide degradation products

Gliadin degradation reactions were performed in duplicate for PSP464 activity on the 11-mer and PSP692 activity on the 33-mer at time points of 30 min, 1 h, 2 h, and 4 h. Following incubation, duplicate reactions were pooled prior to downstream processing to minimize technical variability. Reactions were quenched by the addition of trifluoroacetic acid (TFA) in water to a final concentration of 0.1% (vol/vol). To remove intact enzyme and retain low-molecular-weight degradation products, samples were centrifuged through Microcon centrifugal filter units with a 10 kDa MWCO at room temperature for 30 min. The filtrate, containing peptides <10 kDa, was collected and subjected to C18 ZipTip protocol for desalting prior to LC-MS/MS analysis. The elution solvent for desalting was 50% acetonitrile with 0.1% formic acid in water.

Samples were analysed on a Bruker IMPACT HD mass spectrometer in HRMS mode. MS scans were acquired over an m/z range of 50–3,500 at high resolution in positive ion mode. Raw data were processed in MZMine v3.9 (38), and the fragment list was exported as an Excel list followed by manual annotation. Chromatograms were generated for identified gliadin-derived peptides across the time course.

### Cell culture maintenance and assay method

CaCo-2 cell line was obtained from the repository of the National Centre for Cell Science, Pune. Complete culture medium was composed of DMEM supplemented with 10% FBS and 1% non-essential amino acids and incubated in a humidified chamber at 37°C with 5% CO_2_. All media components were purchased from Gibco, Thermo Fisher Scientific, Inc. Cultures were split in a 1:3 ratio once every 48–72 h, i.e., when ~80–90% confluence was achieved. For assays involving measurement of cytokine secretion and gene expression levels, cells were seeded at an initial density of 5 × 10^4^ cells/well of a 24-well culture plate and maintained in 1 mL complete medium for at least 72 h prior to the assay. For the monolayer permeability assay, cells were grown on a membrane for a minimum of 28 days until transepithelial resistance of each well was a minimum of 500 Ω/cm^2^. During this 28-day culture period, the medium was replaced once every 2 to 3 days. For all cell-based assays, peptides were digested with enzyme in respective reaction buffers for 4 h at 37°C prior to incubation with cells for 45 min. On the day of the assay, cell culture medium was aspirated and replaced with culture medium (control), 1 mg/mL of undigested 33-mer (LQLQPFPQPQLPYPQPQLPYPQPQLPYPQPQPF) or 11-mer (GPQQSFPEQEA), or 1 mg/mL of peptides digested with PSP692 or PSP464 in a 1:500 molar ratio. Synthetic peptides were purchased from Genetoprotein Pvt. Ltd. at a purity >95%, as verified by HPLC.

### Measurement of IL-6 secretion

Previously collected culture supernatant was thawed (not more than one freeze-thaw cycle) and used for IL-6 estimation in pg/mL by ELISA, following the manufacturer’s' instructions (Cat. No: 900-M16 Human IL-6 Mini ABTS ELISA Development Kit, Mfg: PeproTech, Inc.). Readings were recorded at 405 nm and 650 nm at 10-minute intervals for a total time of 40 min. The time point where readings of the assay standard displayed maximum linear correlation between concentration and absorbance was used.

### Quantitation of ZO-1 and occludin transcript levels

The effect of 33-mer and 33-mer digested by PSP464 and PSP692 on ZO-1 and OCLN mRNA expression was investigated by real-time PCR (qRT-PCR) based on the SYBR Green method. Primer sequences were designed in-house and are listed in Table 1, and primers were purchased from IDT, Inc.

**TABLE 1:** Sequences of primers designed in-house for RT-qPCR

Primer name	Sequence (5′ to 3′)
ZO-F	CCAGCATCATCAACCTCTGC
ZO-R	CATGCGACGACAATGATGGT
OCLN-F	CCAATTTATACACCTGCAGCTACT
OCLN-R	GGGGGATCCACAACACAGT
GAPDH-F	TCAAGGCTGAGAACGGGAAG
GAPDH-R	CGCCCCACTTGATTTTGGAG

GAPDH was utilized as an endogenous control. Cells were exposed to either 1 mg/mL 33-mer gliadin/11-mer (PDB ID: 5KSB_I) epitope or to peptides of the same concentration pre-digested with purified enzymes, either PSP464 or PSP692. The incubation period of the assay was 45 min, at the end of which cells were harvested with 0.25% trypsin-EDTA, washed with sterile 1× PBS, and resuspended in equal volumes of Trizol (Thermo Fisher Scientific Inc.) for RNA extraction, following the manufacturer’s protocol. The quality and quantity of total RNA were detected using a NanoDrop spectrophotometer. Equimolar RNA was used to synthesise cDNA using the Verso cDNA kit (Thermo Fisher Scientific Inc.). SYBR Green Master Mix (Thermo Fisher Scientific Inc.), along with cDNA and primers, was used to perform qPCR, and the 2^−∆∆Ct^ method was used to interpret the results.

### Measurement of monolayer permeability

Transepithelial electrical resistance (TEER) was used to monitor the integrity of the epithelial monolayer and was determined using a Millicell ERS-1 volt-ohm meter according to the manufacturer’s instructions. TEER values were calculated as ohms × cm^2^. Monolayers reaching TEER values >500 Ω·cm^2^ were considered to have an appropriate barrier function and were used for further study. Paracellular permeability was measured as FITC-dextran 4 kDa (SRL Pvt. Ltd.) fluorescence intensity on the apical side.

### Quantification of ZO-1 expression at CaCo-2 cell surface

Changes in expression of ZO-1 at the cell surface were determined by confocal microscopy. Cells were incubated for 45 min with 33-mer only or enzyme-digested 33-mer; cells for microscopy were grown in 12-well removable chamber slides (Ibidi Pvt. Ltd.). At the end of the incubation period, cells were washed with 1× PBS and fixed in 4% paraformaldehyde in 1× PBS for 10–15 min. This was followed by washing with 1× PBS, after which blocking was done for 1 h at room temperature in 5% BSA. Then, 100 µL of a 1:2,500 dilution of anti-ZO1-AlexaFluor647 (Thermo Fisher Scientific Inc.) in 3% BSA in 1× PBS was added to each sample well and incubated overnight at 4℃ in the dark. The antibody solution was aspirated, slides were washed with 1× PBS, and a 1:10,000 dilution of DAPI was added to each well 10–15 min prior to washing, followed immediately by addition of 90% glycerol as mountant. Cells stained with DAPI only were used as negative control. Whole-field integrated density of fluorescence for 10 random fields per sample was determined using ImageJ software (39).

### Statistics

Data were analyzed with GraphPad v8.3 (San Diego, CA) software. One-way ANOVA and Tukey’s post-test were used to determine statistical significance. A *P*-value of <0.05 was considered significant. Data are expressed as means ± standard deviation (SD) of triplicate samples for all assays performed.

## RESULTS

### Bioinformatic analyses and *in silico* screening

From the bioinformatic analysis of the metagenome data set PRJNA757365 (Fig. 1), nine bacterial species were found to be differentially abundant between celiac and non-celiac samples, viz., *Faecalibacterium prausnitzii*, *Segatella copri*, *Eubacterium rectale*, *Bacteroides uniformis*, *Bacteroides fragilis*, *Bifidobacterium adolescentis*, *Bifidobacterium longum*, *Blautia wexlerae*, and Lachnospiraceae bacterium (with a normalized relative abundance >1) (Supplementary ST1A and ST1B). Over 200 proline-specific peptidases (PSPs) from these nine bacterial species and from peptidase databases that fit our inclusion/exclusion criteria underwent *in silico* screening.

After application of selection criteria to candidates from peptidase databases and metagenome data analysis, a total of 20 protein sequences were predicted by InterPro (40) to contain PSP domains. The top-ranked models from AlphaFold2 (31) tertiary structure prediction for all 20 protein sequences (ST1C) were docked with all seven gliadin epitope ligands using the HPepDock (34) and HDock (41) servers in site-specific docking mode. Based on (i) formation of a donor hydrogen bond of length <3 Å by catalytic serine with a peptide bond of the ligand (ST1F), (ii) MM/GBSA of the docked structure (ST1D), and (iii) ligand RMSD, protein-ligand contacts, ligand-protein contacts, and percent occupancy of catalytic residues determined from MD simulation (ST1E), PSP464 and PSP692 sequences were identified as promising candidates for experimental validation.

**Fig 2:**
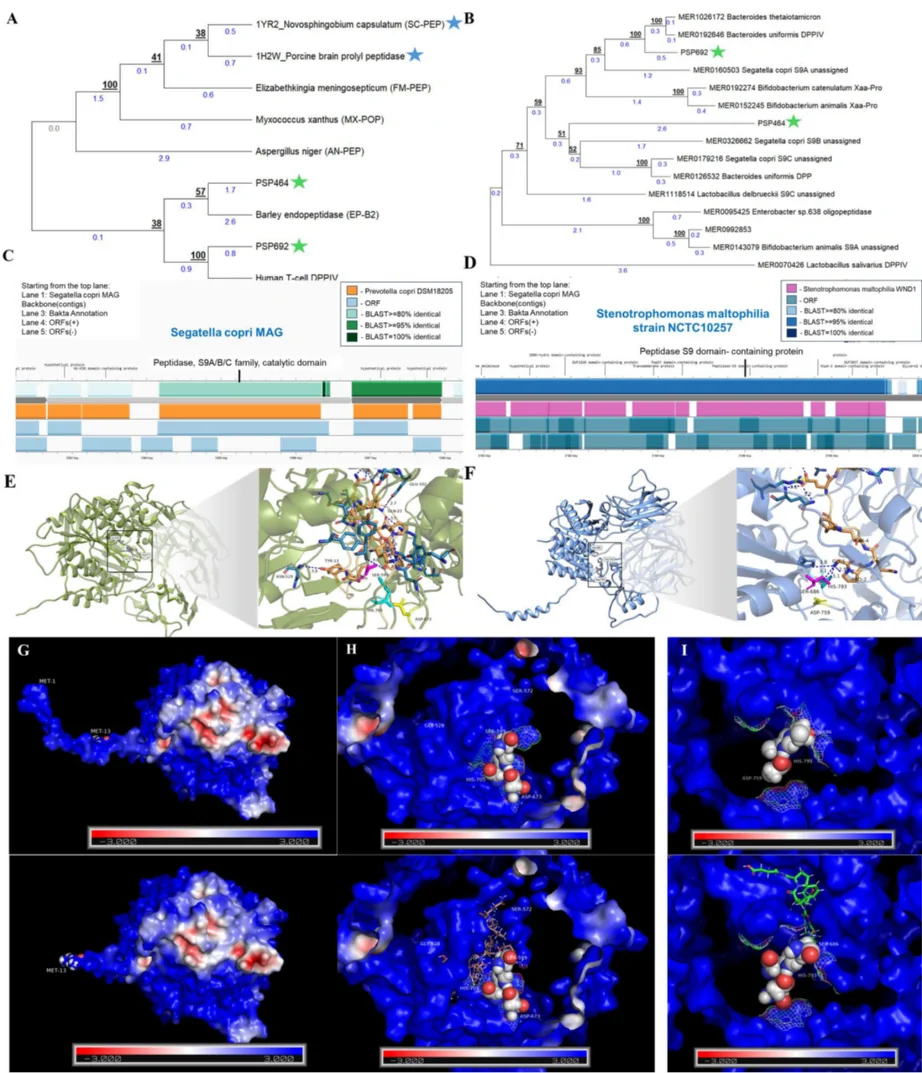
(**A**) A phylogenetic tree constructed from query sequences (green star) of PSP464, PSP692, along with experimentally validated gluten-degrading enzymes (GDEs), GDEs with PDB structures (blue star), and metagenome-derived PSPs that are annotated in MEROPS but not validated. For metagenome-derived PSPs, bacteria from the top 10 genera of the core human gut microbiome in UHGG (45), annotated to be part of a healthy human gut per HumGut (46), were considered. (**B**) De-duplicated and merged bins annotated as *Segatella copri* by CheckM (22) were used to construct a genomic map annotated by the Bakta module of Proksee (47). The location for PSP692 in the reconstructed genome was determined by BLAST of contigs annotated by Bakta (48) as S9/S8/Xaa-Pro peptidase. (**C**) The genome of *Stenotrophomonas maltophilia* NCTC10257 was downloaded from NCBI and used to construct a genome map in Proksee (47); this was then aligned to the genomic standard strain *S. maltophilia* WND-1. (**D**) Docking of PSP692 with 33-mer gliadin and a zoomed-in view shows the predicted active site: Ser599 (magenta), Asp673 (yellow), and His705 (cyan). Hydrogen bond interactions for docking of PSP692 with each of the tested ligands, along with length of the H-bond and binding energy, are tabulated (S4a). (**E**) Docking of PSP464 with 11-mer gliadin and a zoomed-in view shows the predicted active site: Ser686 (magenta), Asp759 (yellow), and His793 (cyan). Hydrogen bond interactions for docking of PSP464 with each of the tested ligands, along with length of the H-bond and binding energy, are tabulated (S4a). (**F**) Putative sites of ligand cleavage upon docking with PSP464 or PSP692, based on bond torsional energies determined from molecular dynamics simulation in Desmond. (**G**) Top: Exterior view of APBS of PSP692 at pH 6. Bottom: Exterior view of APBS of the PSP692-33-mer docked complex at pH 6. (**H**) Top: View of the active site of PSP692 at pH 6; surface color is based on electrostatic charge distribution. Bottom: APBS of the active site of PSP692 docked with 33-mer gliadin at pH 6. (**I**) Top: View of the active site of PSP464 enzyme at pH 4. Bottom: View of the active site of PSP464 docked with 11-mer gliadin at pH 4. Results of PLIP evaluation of PSP692-33-mer and PSP464-11-mer docked structures are present in Supplemental material at ST2F. View tuples for PyMOL visualizations are present at ST2G.

A phylogenetic tree was constructed in MEGA12 (42) to compare our recombinant peptidases with bacterial peptidases that are validated to have gluten-degrading activity (Fig. 2A) and with various peptidases predicted from metagenomic data and annotated in MEROPS (29) (Fig. 2B). The bootstrap values were calculated from 500 replicates. All docking results were visualized in UCSF Chimera 1.19 (43). MM/GBSA was determined using HawkDock server (44). PDB2PQR and APBS functions were done using Chimera plug-ins. APBS result files were visualized in PyMOL. To enable direct structural comparison, all molecular visualizations generated in PyMOL were aligned and displayed using identical view tuples (ST2G), ensuring uniformity in the spatial presentation of active sites and binding regions. Similar data analysis was applied to PRJNA486782; however, none of the metagenomes obtained were of sufficient completeness to be suitable for further screening.

We found that the gene encoding PSP692 from the metagenome reconstructed genome of *S. copri* has an identity of 80–85% to the reference genome of *S. copri* DSM18205. The gene coding for PSP464, determined from the whole-genome submission for *S. maltophilia* NCTC10257 (GenBank: GCA_900186865.1), is >90% identical to a gene from *S. maltophilia* WND-1. We further determined from phylogenetic analysis of protein sequences of PSP464 and PSP692 that our query peptidases cluster separately from previously validated gluten-degrading enzymes. Sequences used for construction of phylograms are present in File S1. The clade formation observed in Fig. 2B indicates that PSP sequences group according to enzyme class rather than source organism. This is exemplified by PSP692, derived from *S. copri*, which clusters with DPPIV from *B. thetaiotaomicron* rather than with other peptidases from *Segatella*. Thus, despite belonging to the S8/S9 class of enzymes, proline-specific peptidases exhibit subclass-dependent substrate specificity, which underlies the differences in their ability to degrade gliadin.

### Effect of PSP692 and PSP464 on gliadin-induced cytokine secretion and loss of barrier integrity

#### Determination of enzyme kinetics

Upon purification of recombinant enzymes by Ni-NTA affinity method, the enzyme kinetics and post-proline cleavage ability were determined using two synthetic substrates, viz., Z-GP-pNA and Z-PPF-pNA, at variable concentrations. Reactions for PSP464 were carried out at 37°C in pH 4 glycine-HCl buffer, while reactions for PSP692 were carried out in pH 6 PBS buffer at the same temperature.

**Fig 3:**
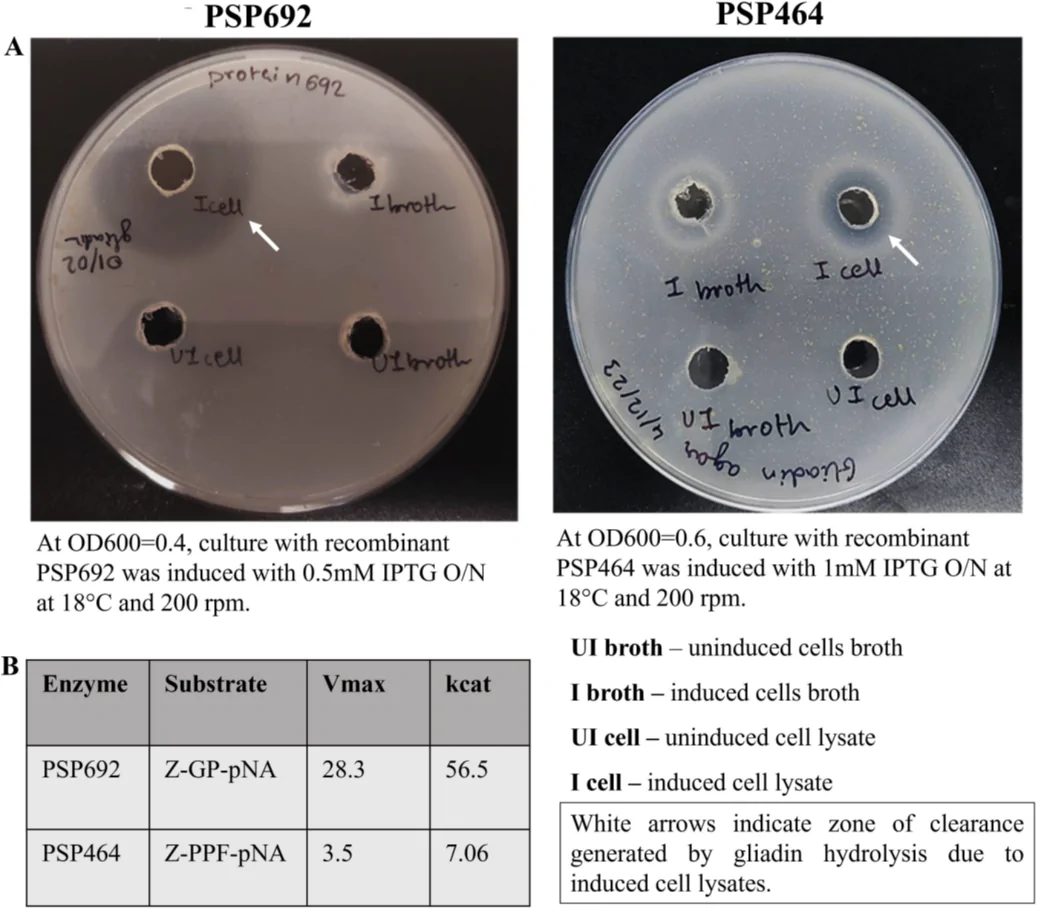
(**A**) The retention of native gliadin-degrading activity by recombinant PSP692 and PSP464 was determined qualitatively by plating 200 μL of induced cell lysate in wells made in gliadin agar plates. The uninduced cell lysate was plated as a control. The agar plates were incubated overnight at 37°C, and as indicated by white arrows, a zone of clearance shows that recombinant proteases are soluble and able to degrade gliadin. Some gliadin-degrading activity is also observed in the broth of induced PSP464 culture, indicating that it is secreted. (**B**) The catalytic efficiency (kcat) for both PSP692 and PSP464 for synthetic peptides modified at the C-terminus with p-nitroanilide was observed from a linear fit of velocity vs substrate concentration profile, as no saturation was observed up to 1 mM substrate. The absorbance was quantified at 405 nm as described in Materials and Methods. The rate of product formation was determined with a standard curve of p-nitroanilide in the respective reaction buffers of PSP692 and PSP464.

The kcat values for PSP692 and PSP464 against the chromogenic gluten motif analog, as calculated from a velocity versus substrate profile over 200–1,000 μM substrate, were found to be 56.5 s⁻¹ and 7 s⁻¹, respectively (Fig. 3B). The kcat value for PSP464 indicates a low turnover rate, while PSP692 demonstrates an average to above-average catalytic rate based on values reported for other non-mutated prolyl oligopeptidases.

#### LC-MS analysis of gliadin peptide degradation products

**Fig 4:**
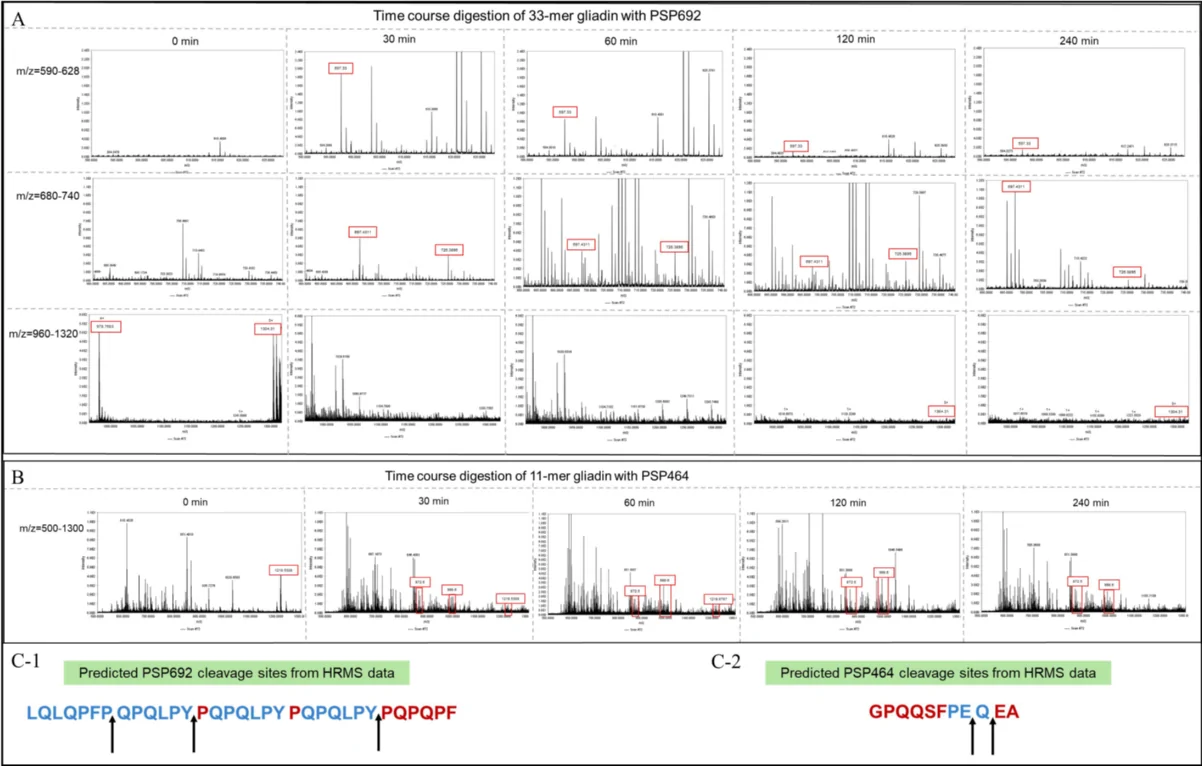
(**A**) Spectra are shown for the undigested control of 33-mer and successive digestion by PSP692 at time points of 30, 60, 120, and 240 min. In the bottom panel, the intact peptide is detected in the control sample as characteristic multiply charged ions at m/z 978 (4+) and m/z 1,304 (3+), consistent with values reported in the literature for the gliadin 33-mer. In digestion samples, the intensity of these parent peptide signals is markedly reduced or below the detectable limit, indicating degradation of the intact peptide upon incubation. Fragment ions appear in the m/z 590–740 range, consistent with the formation of shorter peptide fragments from the proline-rich sequence. Prominent fragment peaks highlighted in the spectra include m/z 597, 697, and 725, which are observed in digestion samples but are absent or present in the undigested control. Based on their masses and the sequence composition of the 33-mer peptide, these ions likely correspond to peptide fragments derived from the N-terminal proline-rich motifs, including LQLQP (~597), QPFPQ (~625), and QLQPFP (~725). The ion at m/z ~ 697 likely represents an intermediate fragment derived from related sequence regions within the repetitive QP-rich motif. The relative intensities of several fragments decrease over extended incubation, suggesting that these ions correspond to intermediate degradation products that undergo further enzymatic cleavage over time. For the analysis, polyethylene glycol (PEG) ladder ions arising from solvent contamination were identified and excluded (corresponding to m/z values 576, 620, 664, 708, 840, and 884), ensuring that the annotated peaks correspond to peptide-derived species. (**B**) The 11-mer gliadin is detected at expected m/z ~1,218 with charge state +1. The intensity of the parent peak is reduced at all digestion time points; also, two prominent ions at m/z 998 and 872 were observed exclusively in the digestion samples and were absent in the undigested control. These masses correspond to truncated forms of the 11-mer peptide GPQQSFPEQEA, consistent with sequential C-terminal cleavage generating GPQQSFPEQ and GPQQSFPE. **C1–2**) The m/z values of digestion products were mapped to determine the likely sequence of fragment peaks. From this information, putative cleavage sites by action of PSP692 on 33-mer and of PSP464 on 11-mer are represented.

High-resolution mass spectrometry (HRMS) was used to monitor the degradation of the immunogenic 33-mer gliadin peptide (LQLQPFPQPQLPYPQPQLPYPQPQLPYPQPQPF), following incubation with PSP692 (Fig. 4A). In the spectrum of the undigested peptide, signals corresponding to the intact peptide were observed at m/z 978 (4+) and m/z 1304 (3+), consistent with previously reported charge states for the gliadin 33-mer in ESI-MS analyses (49). In contrast, these parent peptide signals were markedly reduced or below the detectable limit in all digestion spectra, indicating rapid loss of the intact peptide upon incubation. Comparison of spectra obtained from the undigested peptide and digestion time points revealed the progressive appearance of several fragment ions in the m/z 550–750 range, consistent with cleavage of the proline-rich peptide into shorter oligopeptides.

**Fig 5:**
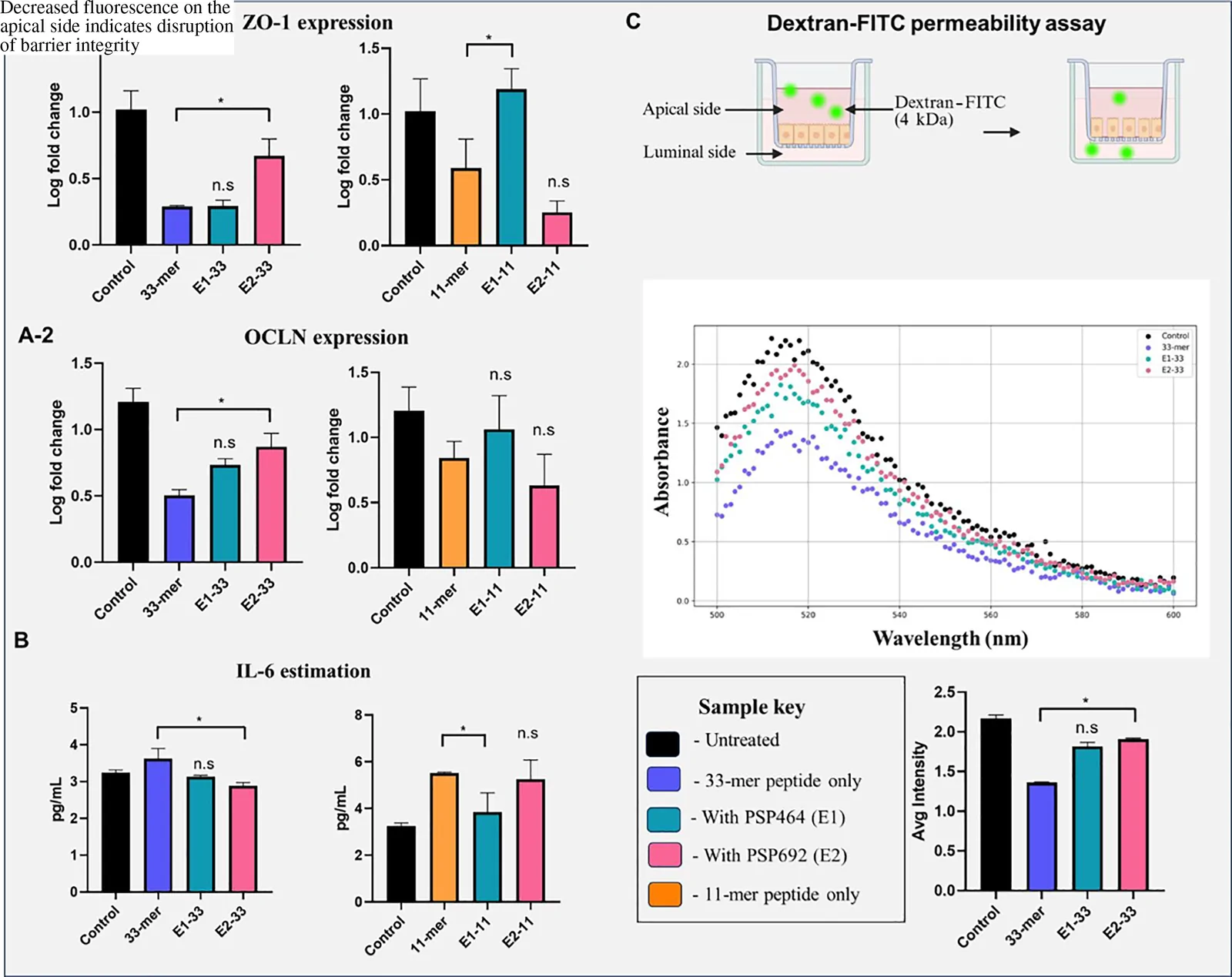
(**A-1** and **A-2**) The levels of ZO-1 and occludin (OCLN) transcripts in cells incubated for 45 min with undigested and enzyme-digested 33-mer and 11-mer gliadin epitopes. PSP692 is able to degrade 33-mer to an extent that significantly restores ZO-1 and OCLN expression, while PSP464 is able to degrade the 11-mer to an extent that significantly restores ZO-1 expression. (**B**) Secretion of IL-6 into culture supernatant was determined by sandwich ELISA; following the manufacturer’s instructions, the difference in absorbance at 650 nm and 405 nm was used to calculate IL-6 concentration by extrapolation from a standard curve. PSP692 degraded 33-mer, while PSP464 degraded 11-mer gliadin to an extent that results in a significant decrease in IL-6 secretion. (**C**) Barrier function of CaCo-2 cells cultured on Transwell membrane for 28 days was determined as a function of 4 kDa FITC-dextran fluorescence in the apical chamber (measurement range of 500–600 nm). Disruption of barrier integrity leads to loss in signal intensity. Treatment of 33-mer with PSP692 prevents barrier disruption. For all assays, reactions were performed in triplicate; statistical significance (*P < 0.05) was determined using one-way ANOVA and Tukey's post-test. Error bars represent mean ± SD.

Among the detected ions, three fragment peaks at m/z 597, 697, and 725 were consistently observed in spectra at all time points of digestion but were absent in the undigested control, indicating that they arise from enzymatic processing of the 33-mer peptide. These fragments appeared early during incubation and were detectable across all digestion time points examined. Notably, the signal intensities of m/z 597 and 725 gradually decreased with increasing incubation time, suggesting that they represent primary degradation products generated during the initial stages of cleavage and subsequently further processed into smaller peptides. The observed m/z values fall within the expected mass range for 5–6 amino acid fragments, consistent with cleavage of the repetitive, proline-rich motifs present in the 33-mer sequence.

In addition to these fragments, a peak at m/z 697 was also detected. This ion was also absent in the undigested peptide sample and present at all time points of digestion. This peak likely corresponds to an internal fragment with sequence QPQLPY, which occurs thrice in the parent peptide. Its recurrence in the digestion spectra and its mass range, consistent with intermediate peptide fragments, along with increasing intensity over digestion time, suggest that it may be a stable fragment accumulating from enzymatic processing of the 33-mer.

Overall, the emergence of multiple fragment ions in the ~590–740 m/z region, combined with the concurrent decrease in the parent peptide signal (m/z corresponding to the intact 33-mer), supports the progressive enzymatic degradation of the immunogenic gliadin peptide. The detection of transient intermediate fragments that diminish over time further indicates a stepwise cleavage process, in which larger peptide fragments generated during the early stages of digestion are subsequently converted into smaller products as incubation proceeds. Polyethylene glycol (PEG)–like peaks displaying the characteristic ~44 Da spacing were observed in the 30 min and 60 min digestion spectra of the 33-mer peptide. These signals were attributed to instrument carryover, and peaks of Δm/z = 44 ± 2 Da were excluded from fragment assignment.

In the PSP464-digested 11-mer, two prominent ions at m/z 998 and 872 were observed exclusively in the digestion samples and were absent in the undigested control. These masses correspond to truncated forms of the 11-mer peptide GPQQSFPEQEA, consistent with sequential C-terminal cleavage generating GPQQSFPEQ and GPQQSFPE. The stepwise decrease in mass supports a successive digestion mechanism.

#### Expression of ZO-1 and occludin

ZO-1 is a critical component of intestinal tight junctions, functioning as a platform for the attachment of occludin and claudins at intercellular sites and for adherence of the actin cytoskeleton on the cytoplasmic side. Overall, this assembly plays a pivotal role in the regulation of barrier function. The CaCo-2 cells, upon reaching 80–90% confluence, were exposed to either 1 mg/mL of undigested or enzyme-digested peptide. After exposure to gliadin epitopes for 45 min, the RNA was harvested and immediately used to synthesize cDNA. From the qPCR results, we observed that PSP464 was able to significantly degrade the 11-mer epitope, as evidenced by the restoration of ZO-1 transcript levels. Similarly, PSP692 was able to significantly degrade the 33-mer peptide, leading to restoration of the transcript levels of key barrier proteins, viz., ZO-1 and occludin (Fig. 5A1-2). These findings suggest that PSP464 and PSP692 may play a role in ameliorating barrier function by reducing the immunogenic effects of gliadin-derived peptides.

#### Inflammatory cytokine estimation

The secretion of IL-6 has been well established as an important marker of gut inflammation based on celiac patient duodenal biopsy-derived organoids. At a 1:500 molar ratio of enzyme: peptide, PSP692 and PSP464 were able to degrade the 33-mer and 11-mer respectively (Fig. 5B), to an extent that significantly reduces IL-6 secretion into the culture supernatant. These results provide evidence that the peptidases have a direct effect on mitigating the inflammatory response induced by gliadin and its derived peptides, thus potentially offering therapeutic benefits in CeD.

#### Restoration of monolayer barrier integrity

The effect of PSP464 and PSP692 in modulating barrier integrity was estimated based on the flux of a small molecule, viz., FITC-dextran 4 kDa, from the apical to basolateral region of the Transwell. Fluorescence emission from the apical side of the Transwell membrane was determined spectrometrically in the range of 500–600 nm at 1 nm intervals (Fig. 5C). However, readings in the range of 515–525 nm, which correspond with emission maxima of FITC, were considered for statistical comparison. Relative to untreated cells, the addition of the 33-mer peptide to the apical side of the CaCo-2 monolayer resulted in a significant increase in monolayer permeability, as indicated by a decrease in the fluorescent signal of the apical compartment. This demonstrates that the 33-mer peptide compromises the integrity of the epithelial barrier. However, when the 33-mer treated with PSP692 was introduced, the permeability of the monolayer was significantly restored, as indicated by the recovery of the fluorescent signal.

#### Effect of enzyme treatment on surface expression of ZO-1

**Fig 6:**
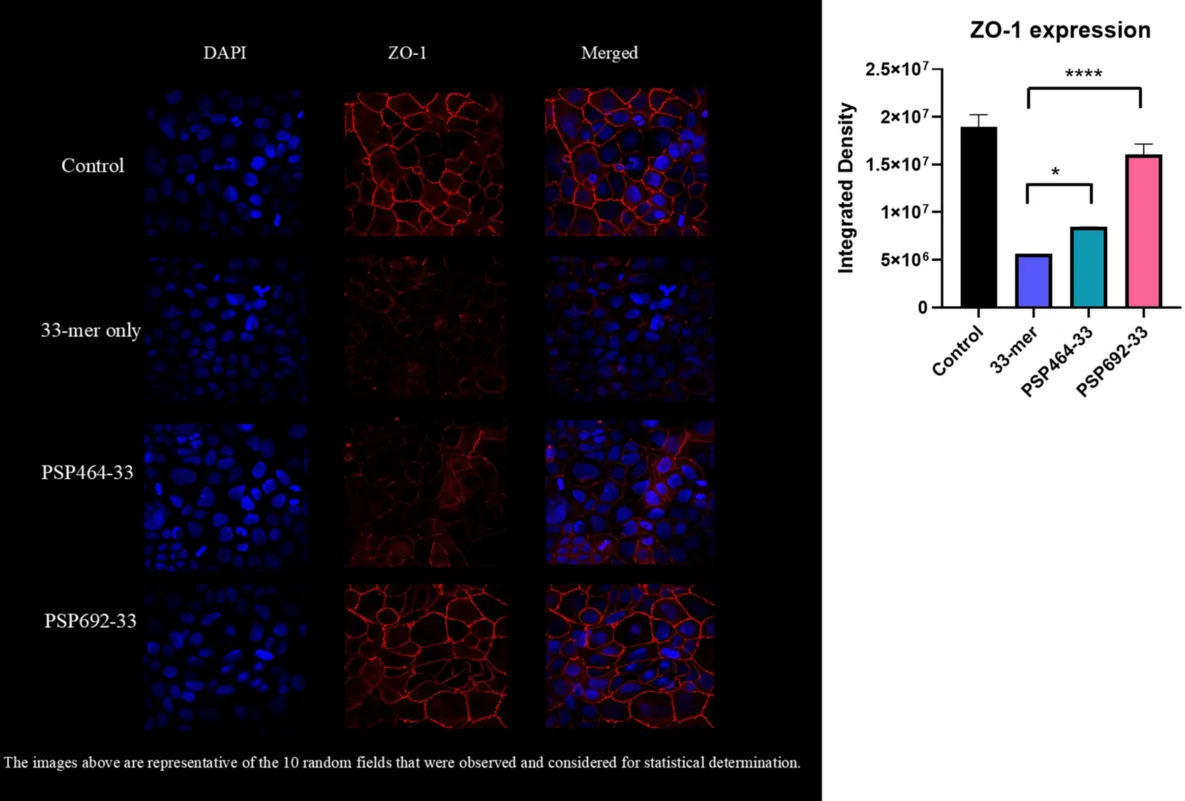
Quantitation of ZO-1 protein at the cell surface was determined by staining with a directly tagged anti-ZO-1 monoclonal antibody. For analysis of ZO-1 expression at the cell surface in 33-mer undigested/pre-digested conditions, ten images of each sample were acquired at random on an Olympus FV3000 at 63× magnification. Images are representative of 10 random fields acquired per sample.

To further investigate the protective effect of PSP692 and PSP464 on CaCo-2 cells, confocal microscopy was employed to visualize ZO-1 protein, a critical component of tight junctions (Fig. 6). ZO-1 is a critical component of intestinal tight junctions, functioning as a platform for the attachment of occludin and claudins at intercellular sites and for adherence of the actin cytoskeleton on the cytoplasmic side. Overall, this assembly plays a pivotal role in the regulation of barrier function. Nuclear staining with DAPI was also used to visualize any potential changes in nuclear morphology due to treatment with recombinant enzymes.

The images for each sample were visualized in ImageJ using the Olympus viewer plugin. Fluorescence signal was determined for the full field. The averaged integrated density of 10 random fields per sample clearly demonstrates a very significant increase in expression of adhesion proteins at tight junction sites when 33-mer gliadin is digested with either PSP464 or PSP692. This result demonstrates that the efficacy of PSP464 and PSP692 in restoring barrier function (by degrading 33-mer) is not restricted to an increase in transcripts of ZO-1 but is also visible at the cell surface.

## DISCUSSION

CeD is an autoimmune enteropathy characterized by chronic intestinal inflammation, initiated and sustained by immunogenic gluten-derived peptides, particularly the proline-rich 33-mer α-gliadin fragment that evades degradation by human gastrointestinal proteases. While host genetic susceptibility, particularly HLA-DQ2/DQ8, plays a necessary role in disease onset (50), not all carriers develop CeD, as evidenced by the absence of 100% concordance of disease development in monozygotic twins (51), indicating the involvement of other environmental or microbial modulators (52).

Traditional efforts in the search for glutenases have largely relied on labor-intensive functional screening of microbial isolates or environmental DNA libraries, which, while useful, are inherently time-consuming and inefficient. These approaches tend to yield a limited number of hits, often favoring enzymes that are abundant, easily expressed, or already well-characterized. In contrast, our approach leverages high-resolution metagenomic data from human cohorts, viz., PRJNA757365 and PRJNA486782 (the only CeD metagenome data sets that are publicly available), processed through a tiered pipeline optimized for both taxonomic and functional fidelity. Rather than relying solely on gene annotations or conserved domain matches, we employed metagenomic assembly (via MEGAHIT) (20), followed by taxonomic binning (MaxBin2) (21) and protein-level functional screening (via RAPSearch2) (25), using the UniRef90 database. This integrated pipeline enabled not only the identification of low-abundance but catalytically relevant genes, but also their linkage to specific taxa with high confidence. This was particularly advantageous in our study, where we were able to resolve strain-level genomes from *Segatella copri* and *Stenotrophomonas maltophilia*, and identify unannotated peptidases that share structural similarity rather than sequence homology with known glutenases. Such structural convergence would have been missed by conventional alignment-based annotation strategies alone. Our method thus facilitated the discovery of functionally relevant yet previously unrecognized prolyl peptidases that are differentially abundant in non-CeD metagenomes, highlighting the power of structure-guided metagenomic mining for therapeutic enzyme discovery. We focused mainly on novel proline-specific peptidases (PSPs) (<30% similarity) enriched in metagenomic bins from non-CeD samples, hypothesizing that their relative abundance might correlate with protective phenotypes.

Phylogenetic analysis revealed that the two candidate enzymes identified in this study, PSP692 from *Segatella copri* and PSP464 from *Stenotrophomonas maltophilia,* cluster distinctly from previously characterized gluten-degrading enzymes which are validated experimentally (Fig. 2B). *Stenotrophomonas maltophilia* is reported as an opportunistic pathogen in certain host conditions. However, in this study, only the gene encoding the enzyme of interest was utilized, and recombinant expression was carried out in *E. coli* BL21DE3. The enzyme itself is not associated with pathogenicity, and its application in our study is independent of the source organism. Despite sharing conserved motifs with known proline-specific proteases, these enzymes do not cluster closely with functionally validated peptidases, highlighting their novelty and the untapped diversity of glutenases within the human gut microbiome. Both enzymes belong to the S9 (subtilisin-like serine proteases) and Xaa-Pro peptidase families, which are broadly implicated in the cleavage of proline-rich substrates but are rarely annotated with specific glutenase function in standard databases. For instance, the S9 family includes a wide spectrum of endopeptidases with overlapping catalytic triads (Ser-His-Asp), while Xaa-Pro dipeptidyl peptidases act at the N-terminal proline of peptides. However, the lack of detailed substrate-specific characterization for many members of these families often limits their therapeutic consideration.

To refine our selection, we integrated enzyme family classification from MEROPS (29) and BRENDA (30) with subcellular localization predictions, prioritizing extracellular enzymes likely to encounter dietary peptides in the gut lumen. AlphaFold2 (31)-based structure prediction enabled the resolution of high-confidence tertiary models for each enzyme, including the orientation of the catalytic triad and substrate-binding pocket. These structures were docked against a panel of immunogenic gliadin peptides, namely the 33-mer (LQLQPFPQPQLPYPQPQLPYPQPQLPYPQPQPF) and shorter epitopes, including 5KSB, 4OZI, and 5IJK, using HPepDock (34). Only those complexes exhibiting catalytically relevant interactions were selected for molecular dynamics (MD) simulations. These structural interaction criteria are well-established predictors of enzyme-substrate interaction (53, 54) and allowed us to prioritize enzyme-substrate pairs with high catalytic plausibility. The identification of PSP692 and PSP464, which had not been previously linked to gluten metabolism, underscores the power of this structure-guided metagenomic mining approach in uncovering novel, therapeutically relevant enzymes.

Following recombinant expression and purification, enzyme kinetics determined using Z-GP-pNA and Z-PPF-pNA, respectively, revealed that PSP692 has a higher catalytic turnover (kcat = 56.5 s⁻¹) compared to PSP464 (kcat = 7 s⁻¹); however, these substrates alone do not provide adequate insight into the ability to degrade native gliadin epitopes.

The functional consequences of enzymatic cleavage were evaluated in a CaCo-2 epithelial cell model, which mimics the intestinal barrier and responds to gliadin-induced epithelial stress (55, 56). In the untreated condition, exposure to the 33-mer (LQLQPFPQPQLPYPQPQLPYPQPQLPYPQPQPF) and 11-mer (GPQQSFPEQEA) led to pronounced epithelial disruption, characterized by a marked increase in IL-6 secretion, decreased mRNA expression of tight junction markers ZO-1 and occludin, elevated monolayer permeability (via FITC-dextran flux), and a loss of ZO-1 protein localization at intercellular junctions, as visualized by confocal microscopy. These changes are in line with known immunopathological responses of intestinal epithelium to gluten-derived peptides in CeD.

Pre-digestion of the 33-mer with PSP692 significantly attenuated these adverse effects. IL-6 secretion was reduced to near-baseline levels (Fig. 5B), and transcript levels of tight junction markers were restored significantly (Fig. 5A1-2). In comparison, PSP464-treated 11-mer also showed a significant reduction in IL-6 levels and restoration of ZO-1, consistent with its action on a shorter epitope. Importantly, both enzymes restored transepithelial barrier integrity as measured by the decrease in FITC-dextran permeability (Fig. 5C), with PSP692 showing superior efficacy, consistent with its preference for the full-length 33-mer.

Confocal microscopy further confirmed these results at the protein level. In the untreated condition, ZO-1 staining was decreased, reflecting junctional disruption. In contrast, both PSP692- and PSP464-digested peptide treatments showed pronounced relocalization of ZO-1 to the cell membrane. Quantitative image analysis revealed a significantly higher fluorescence intensity in PSP692-treated cells compared to PSP464 (Fig. 6), suggesting a more complete restoration of epithelial polarity and tight junction integrity. These findings collectively indicate that while both enzymes mitigate gliadin-induced epithelial injury, PSP692 demonstrates a more potent protective effect, likely due to its higher turnover and capacity to degrade the immunodominant 33-mer.

HRMS analysis provided evidence of direct biochemical confirmation of gliadin cleavage. The generation of multiple persistent peptide fragments in the m/z ~590–740 range is consistent with partial enzymatic degradation of the gliadin 33-mer (Fig. 4A). Prominent ions at m/z 597, 625, and ~725 likely correspond to short oligopeptides derived from cleavage within the proline- and glutamine-rich motifs (e.g., LQLQP, QPFPQ, and QLQPFP), which are characteristic of this sequence. The detection of the ion at m/z ~697, which is observed to accumulate over time points, can be assigned to fragments from the central QPQLPY motif, which further supports cleavage within repetitive regions of the peptide. The observation that several of these fragments decrease in intensity over time suggests that they represent transient intermediates that undergo further proteolysis, indicating a stepwise degradation process. Notably, the majority of detected fragments correspond to peptides of fewer than eight amino acids in length, suggesting that the resulting degradation products are unlikely to retain immunogenic potential, as T-cell stimulatory epitopes in gliadin typically require longer peptide sequences. Collectively, the concomitant loss of the intact peptide signal (m/z 978 (4+) and 1,304 (3+)), together with the emergence of fragments absent in undigested spectra and present across all digestion time points, supports effective enzymatic breakdown of the immunogenic gliadin peptide under the conditions tested. Additionally, PSP464 is able to cleave at the C-terminus of 11-mer gliadin, generating fragments of which m/z 872 may be assigned to the sequence GQQSFPE. Cleavage at this site would destroy the motif that undergoes deamidation upon action by tissue transglutaminase to unmask the PQQ epitope, thereby preventing generation of the immunogenic motif.

The two enzymes characterized in this study, PSP692 and PSP464, exhibit differential but complementary activity profiles. PSP692 demonstrated strong activity against the full-length 33-mer gliadin peptide, which is the immunodominant epitope. In contrast, PSP464 showed more efficient degradation of the shorter 11-mer gliadin fragment. This finding is particularly significant, as most previously studied glutenases are evaluated solely for their activity against the 33-mer, with little attention given to their ability to process the resulting breakdown products, and previous research does not provide a rationale for the selection of the enzymes detailed therein (57–60). Enzymes with complementary substrate-length preferences may act synergistically to enable more thorough and effective degradation of gluten peptides in the gastrointestinal tract. Such a strategy could prevent the accumulation of partially digested, still-immunogenic fragments, which represents a common limitation of single-enzyme therapies. Both enzymes are also active in the pH range of 4 to 6 (ST1H), thereby making them suitable for use in the full stomach/duodenum.

Together, these multi-parameter validations, encompassing cytokine profiling, gene expression, barrier function, and confocal imaging, underscore the biological efficacy of these enzymes in neutralizing gluten toxicity. Moreover, the complementary action of PSP464 on the 11-mer, corresponding to a region within p31-55 of α-gliadin, which, although not recognized by T cells, is able to induce IL-15 secretion and increase intracellular stress by halting maturation of endocytic vesicles (61–63). A layered approach would not only reduce the concentration of intact immunogenic peptides but also limit the accumulation of shorter, still-immunogenic fragments, which are often overlooked in enzyme evaluations.

Gluten is a heterogeneous and structurally complex protein composite found primarily in wheat, barley, and rye, comprising prolamins (gliadins and hordeins) and glutelins. Its immunopathogenicity in CeD stems from the abundance of proline- and glutamine-rich motifs that resist proteolysis by human gastrointestinal enzymes. Notably, several immunogenic peptides, such as the 33-mer from α-gliadin (6), 18-mer (64), 13-mer (65), 9-mer (66), and the 11-mer (15), have been shown to activate gluten-reactive CD4^+^ T cells in HLA-DQ2/DQ8 individuals. Because of this structural and immunological complexity, a single enzyme may be insufficient to achieve complete detoxification. Our findings demonstrate that a data-driven, structure-guided strategy can successfully identify complementary enzymes such as PSP692 and PSP464 that act on immunogenic peptide substrates of different lengths. Although these enzymes show promising activity against model gliadin epitopes *in vitro*, further studies are warranted to evaluate their utility in gluten degradation and their potential to reduce antigenic burden in the context of CeD management as a preventive or adjunctive strategy to reduce inadvertent exposure.

Beyond the two enzymes characterized in this study, the human gut microbiome harbors a far greater diversity of uncharacterized peptidases with potential gluten-degrading activity. Given the complexity of gluten immunopathogenicity, which involves multiple overlapping epitopes and resistant peptide fragments, systematic mining of metagenomic data could yield additional candidates with complementary substrate specificities, broader pH stability, or enhanced catalytic efficiency. However, a key limitation of the present study is the reliance on the limited number of publicly available CeD-associated metagenomes, which constrains the diversity of enzymes that can be identified. The paucity of high-quality, well-annotated metagenomic data sets from CeD and non-CeD cohorts remains a major bottleneck for comprehensive enzyme discovery. Expanding global efforts to generate large, clinically stratified metagenomic resources will therefore be critical for uncovering the full enzymatic repertoire of the gut microbiome and for developing next-generation combinatorial strategies to mitigate gluten immunotoxicity *in vivo*.

Other than its application in CeD, the integrative methodology described here, which combined metagenomic mining, structure-guided *in silico* screening, recombinant expression, and functional validation, provides a broadly adaptable framework for identifying microbiome-derived bioactive proteins. Because the approach does not rely solely on culture-based isolation and incorporates functional prioritization criteria, it can be extended to other immune-mediated and inflammatory disorders driven by dietary antigens in which microbial peptides or enzymes influence host responses. For example, a similar pipeline could be applied to inflammatory bowel disease, food allergies, metabolic disorders, or even neuroimmune conditions, where microbial metabolites and proteolytic products modulate epithelial or immune signaling. The strategy may also facilitate the discovery of enzymes capable of degrading pathogenic protein aggregates, allergenic food components, or pro-inflammatory peptide motifs in diverse disease contexts. Thus, this workflow represents a scalable platform for mining the microbiome for enzymes with therapeutic potential across a wide spectrum of host-microbe interaction-driven diseases.

### Conclusion

The approach described herein, integrating metagenomic mining, structural modeling, docking, and functional assays, offers a robust pipeline for identifying additional gluten-degrading enzymes from human gut microbial communities. Given the diverse array of gluten epitopes and the variability in individual microbiomes, extending this approach to broader data sets may uncover novel enzyme classes with improved specificity, pH stability, or proteolytic efficiency. Future research should aim to establish the *in vivo* efficacy of such enzymes in more complex physiological contexts, including gastrointestinal transit, exposure to host proteases, and interactions with the host immune system. Rather than focusing exclusively on recombinant enzyme supplementation, synthetic biology-based strategies could also be explored. For instance, stable introduction of validated glutenase genes into commensal duodenal bacteria (e.g., *Prevotella* spp., *Faecalibacterium prausnitzii*, or *Lactobacillus* spp.) could enable sustained, host-compatible gluten detoxification at the site of exposure (67). This would align with the long-term goal of a holistic, microbiome-mediated management of gluten immunotoxicity in CeD.

## Supplementary Material

Reviewer comments

## Data Availability

All metagenomic datasets analyzed in this study were obtained from publicly available repositories. Specifically, raw sequencing data were accessed from NCBI BioProject IDs PRJNA757365 and PRJNA486782. No new sequencing data were generated. Supplementary data are provided in File ST1. All other supporting data are available upon reasonable request from the corresponding author.

## SUPPLEMENTAL MATERIAL

Supplemental material spectrum.03214-25-s0001.docx Supplemental material.

OPEN PEER REVIEW reviewer-comments.pdf An accounting of the reviewer comments and feedback.

ASM does not own the copyrights to Supplemental Material that may be linked to, or accessed through, an article. The authors have granted ASM a non-exclusive, world-wide license to publish the Supplemental Material files. Please contact the corresponding author directly for reuse.
