# Supplementary material for: Harnessing gut microbiome enzymes: Segatella copri and Stenotrophomonas maltophilia prolyl peptidases degrade gliadin peptides and improve epithelial barrier function in a celiac disease model
Source: Microbiol Spectr. 2026 May 29;14(7):e03214-25. doi: 10.1128/spectrum.03214-25 (PMC13339814; doi:10.1128/spectrum.03214-25)

Supplementary information

ST1A. Relative abundance of organisms from PRJNA757365 determined using mOTU profiler and visualized with [microbiomeanalyst.ca](http://microbiomeanalyst.ca)


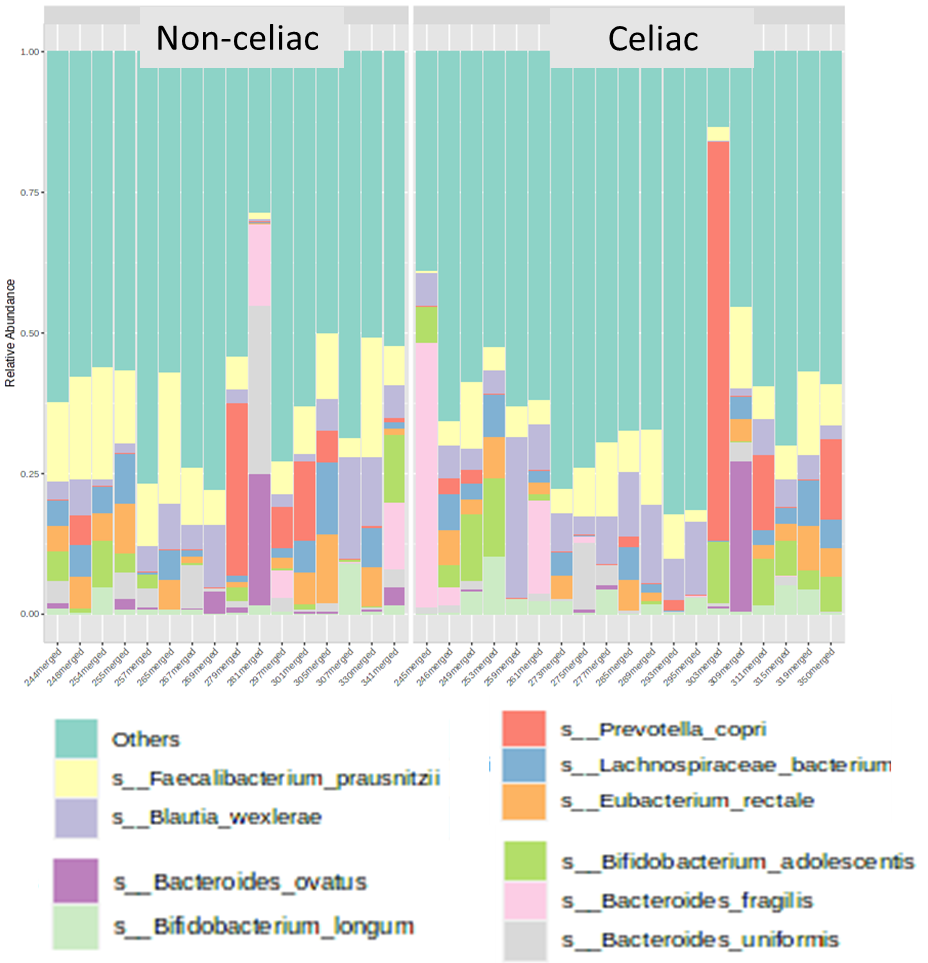


ST1B. Boxplot of top 9 organisms differentially abundant between Celiac and non-Celiac metagenomes


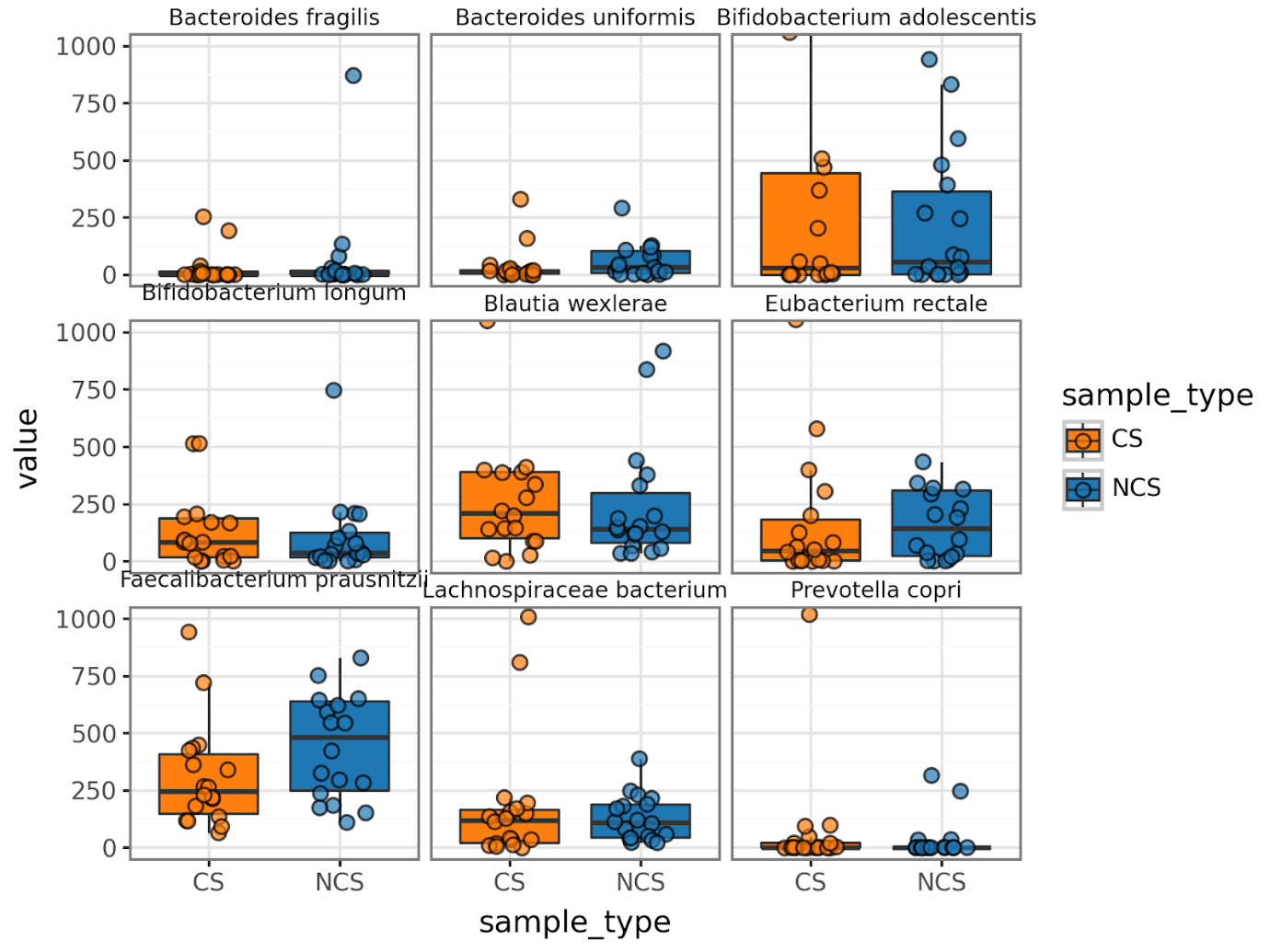


ST1C. Proteins from metagenome analysis and database search that were predicted to be extracellular by a consensus of PrediSi, Phobius and SignalP. Contig sequences containing predicted prolyl peptidases from metagenome analysis are provided in supplementary text file ST1.


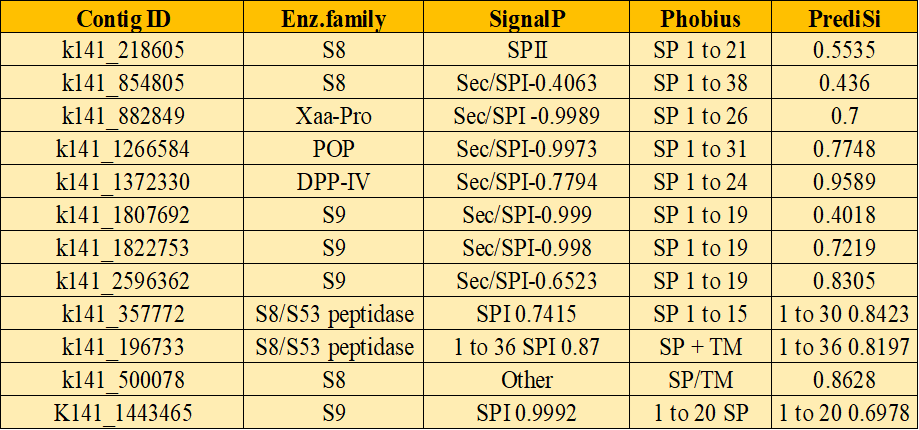


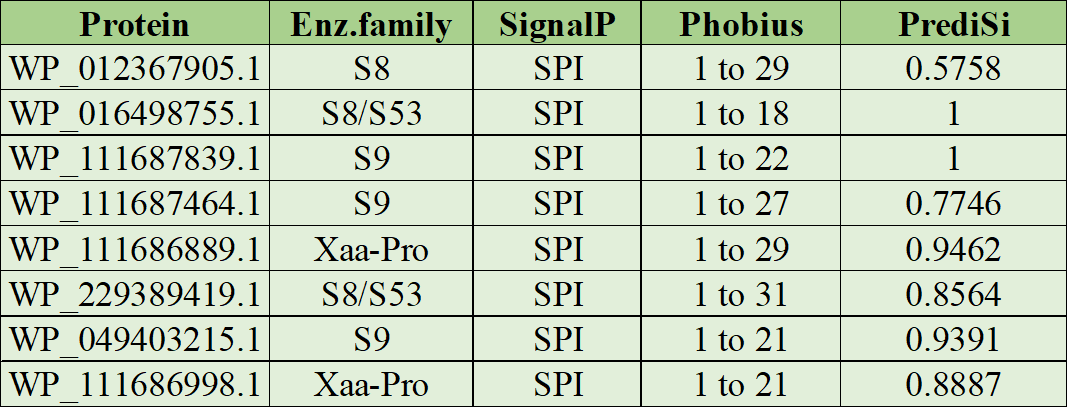


ST1D. MM/GBSA values for PSP464 and PSP692 docked with each of the 7 gliadin epitopes as calculated from


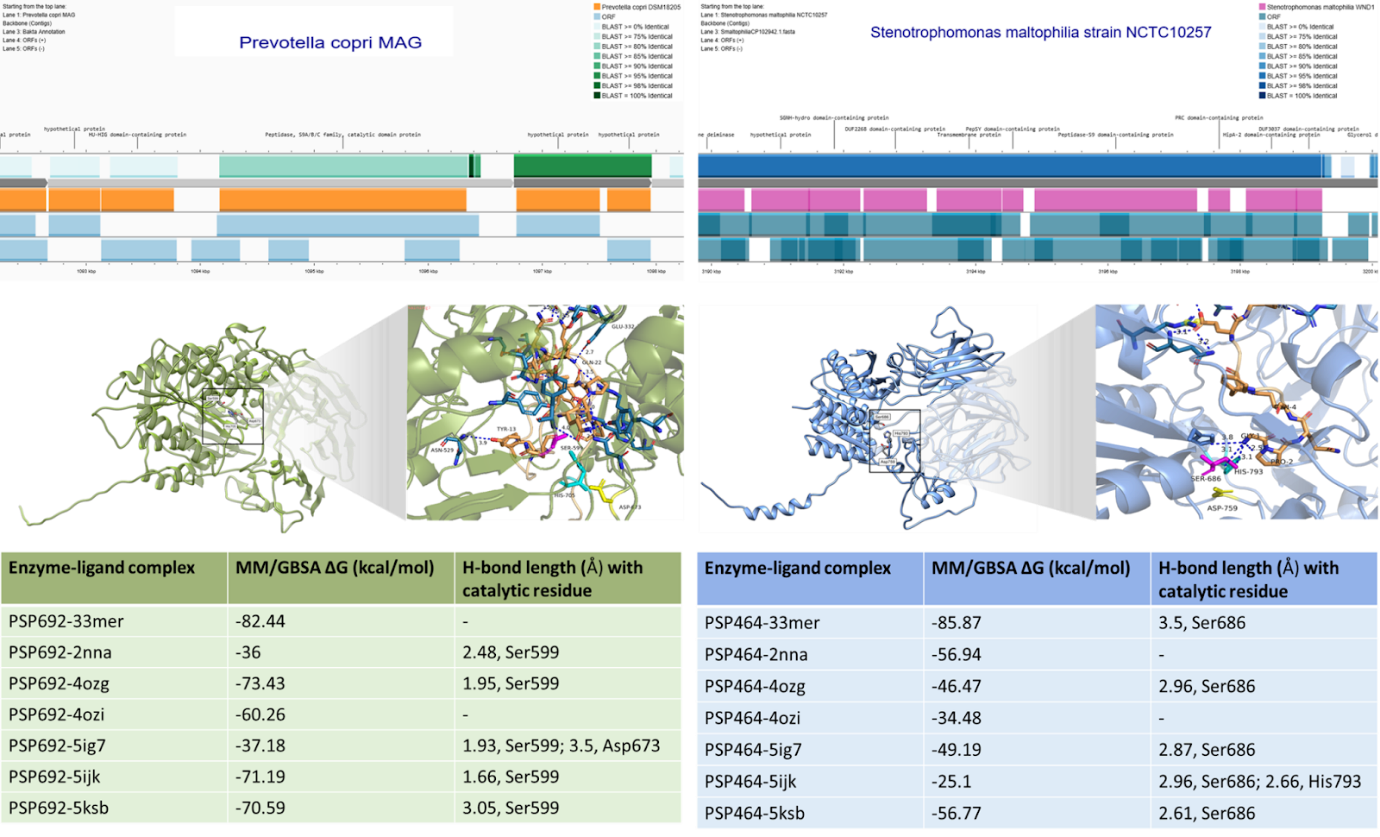


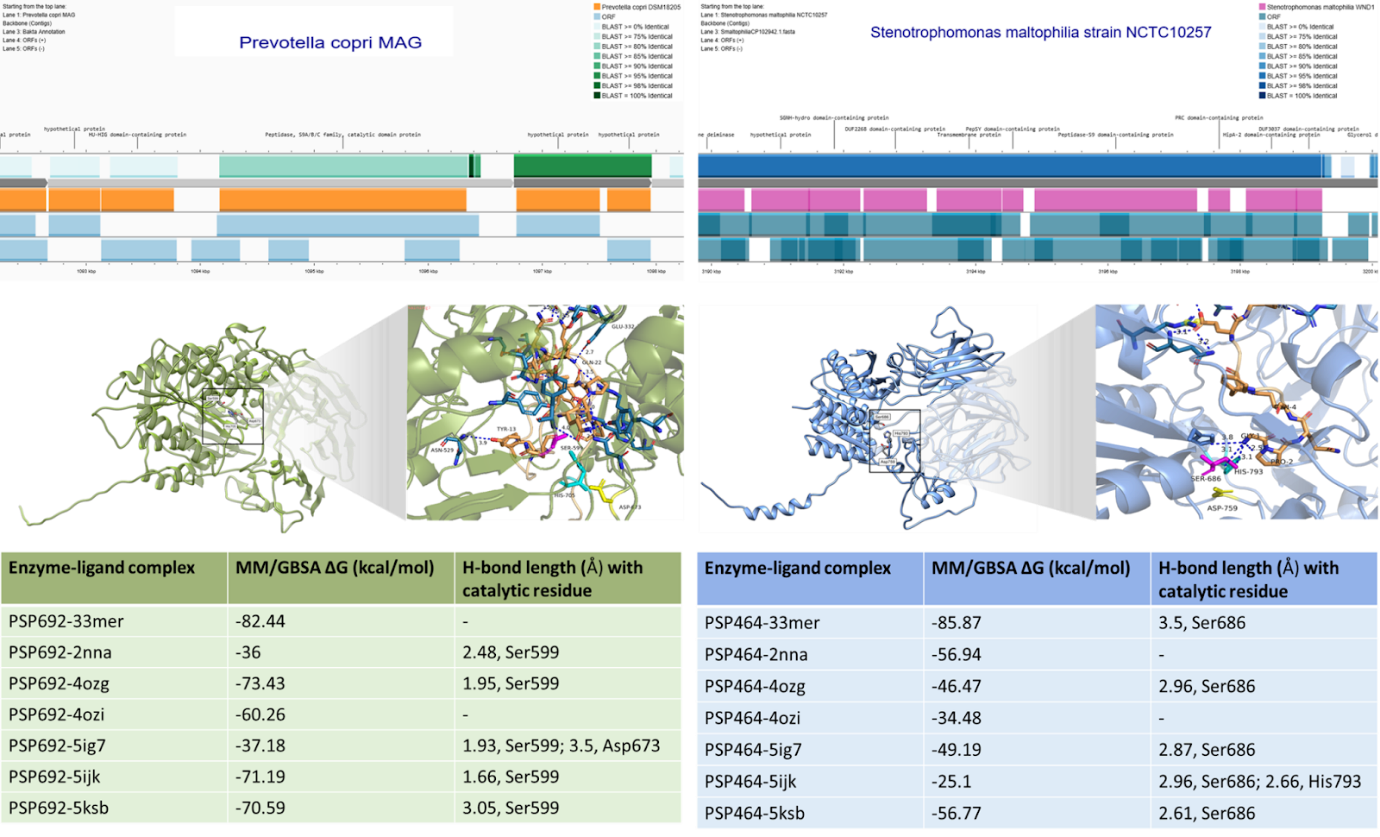


ST1E. PSP464-11 mer ligand RMSD, protein-ligand contacts, percent occupancy per residue and ligand-protein contacts


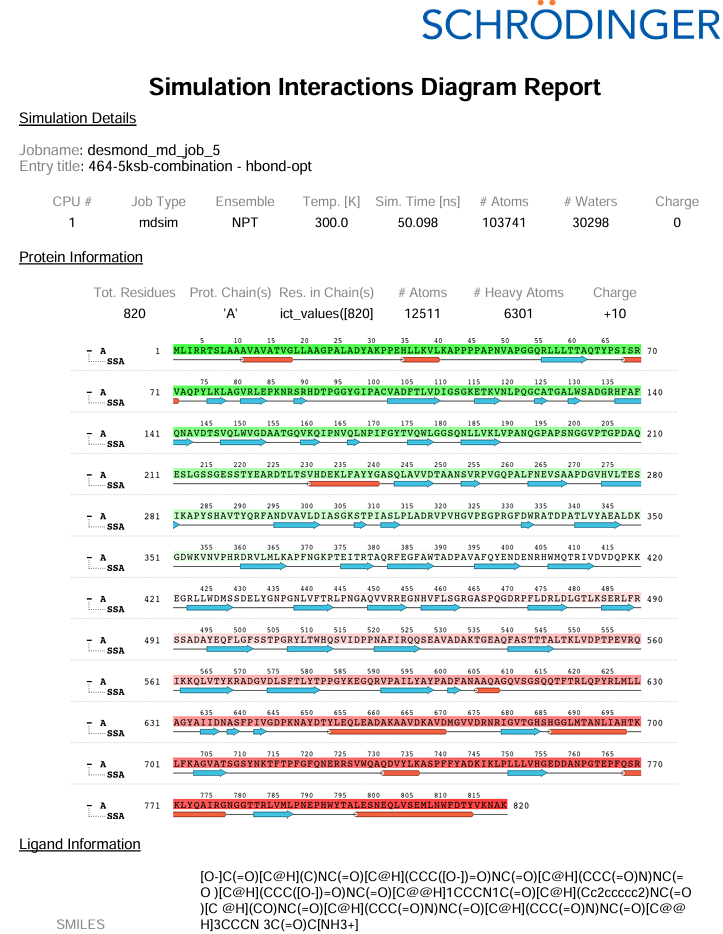


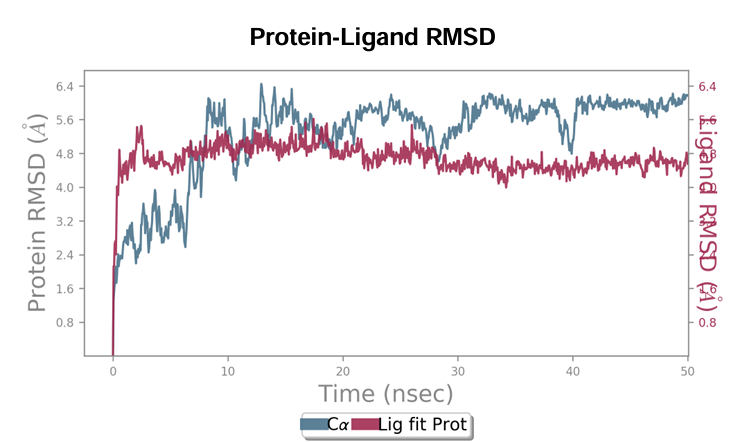


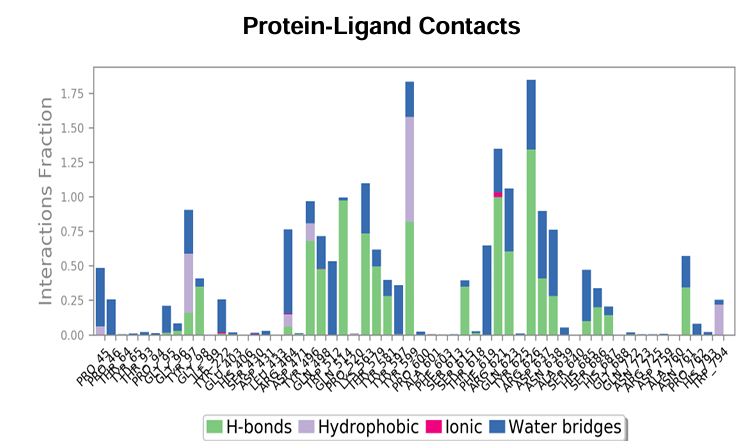


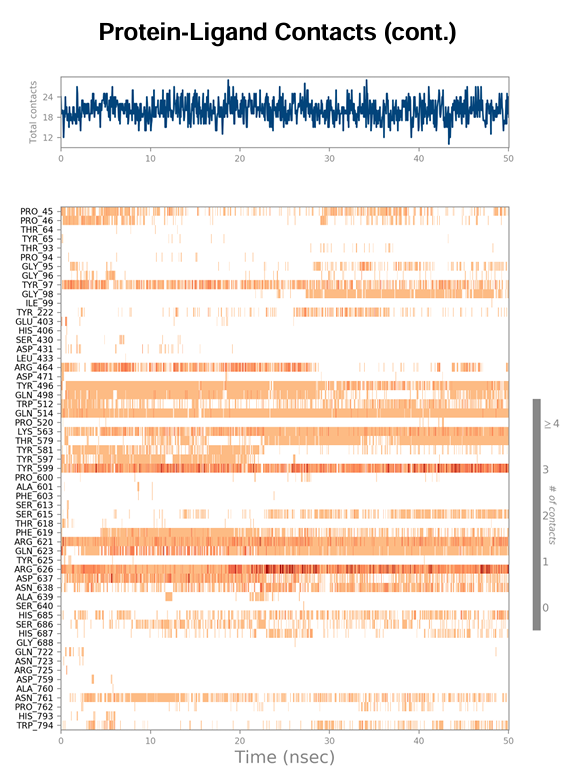


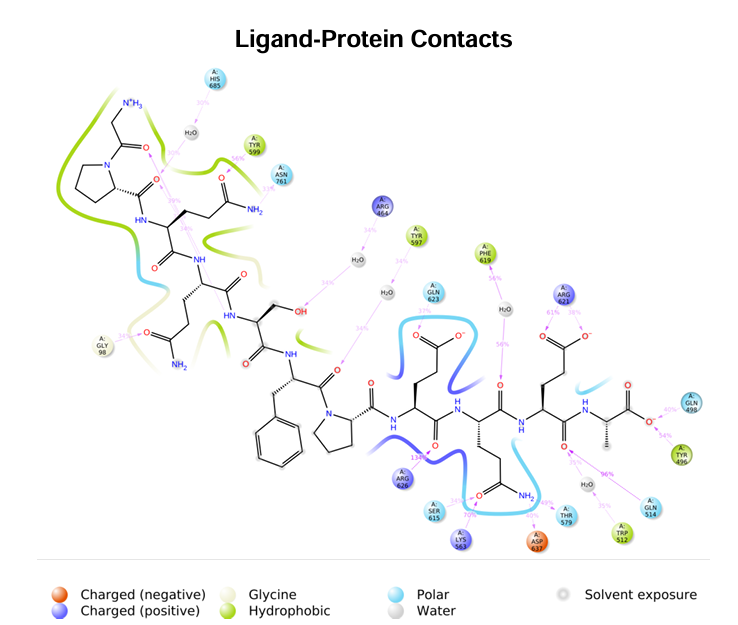


ST1F. Results of docking were analysed with Protein Ligand Interaction Profiler and a major selection criteria was the formation of a donor hydrogen bond between catalytic Serine of enzyme and peptide bond of ligand.

F(i). The predicted catalytic Serine for PSP464 when docked with 11-mer gliadin is predicted to form a donor H-bond with second peptide bond of ligand


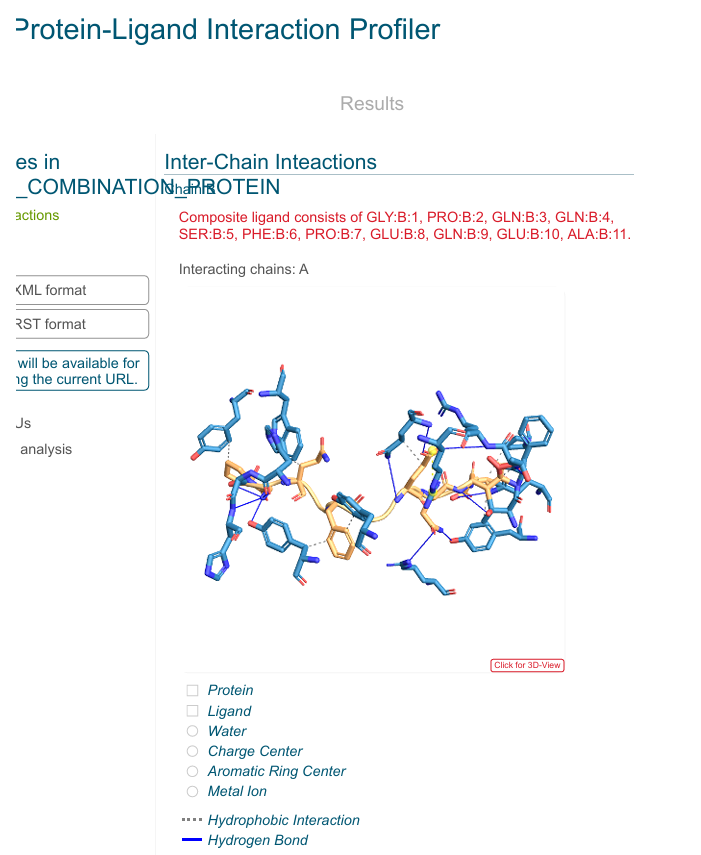

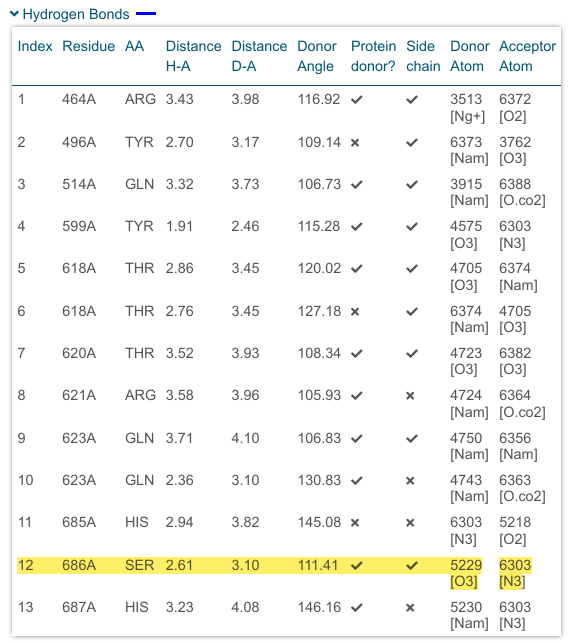


F(ii). PSP692 predicted catalytic serine does not form a H-bond with 33-mer gliadin. However selection of candidate peptidases for MD simulation was based on their ability to form a donor H-bond with a majority of the 7 gliadin epitopes, a criteria that PSP692 satisfies.


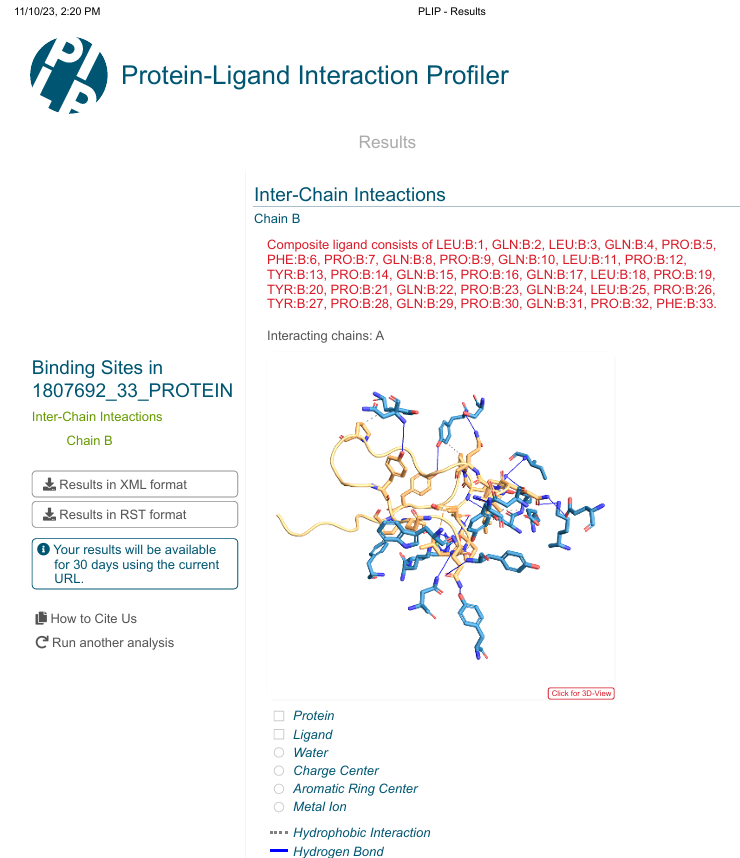


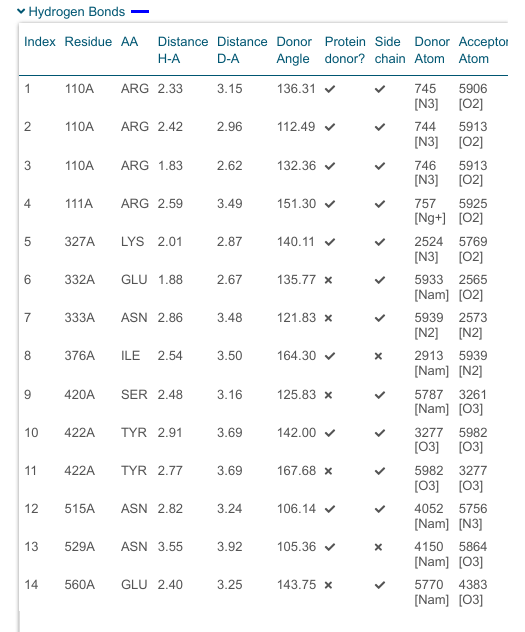


**ST1G. View tuples :**

To observe results from Figure 2, panels G, H and I;first open .pse file in PyMol. Type ‘set view’ in command line, paste the appropriate tuple after the command and press Enter.

**G(i). PSP464 at pH 4 in ligand bound and unbound states**

(-0.5821505188941956, 0.5145198106765747, -0.6295703649520874, -0.8039389252662659, -0.4800156354904175, 0.35108307003974915,

-0.1215655654668808, 0.7105209827423096, 0.6930919289588928,

-5.611218512058258e-06, 0.0001970231533050537, -109.38628387451172, -10.455663681030273, 8.959827423095703, -2.00044584274292,

91.15335083007812, 127.61133575439453, -20.0)

**G(ii). Exterior view of PSP692 at pH 6 in ligand bound and unbound states**

(0.770528197,   -0.177407146,   -0.612217844,

     0.521384001,    0.727935910,    0.445269585,

     0.366662294,   -0.662295282,    0.653394461,

    -0.000000581,    0.000004914, -267.039489746,

    -4.421731949,   -3.469412565,   -0.306641519,

   188.647552490,  345.431793213,  -20.000000000)

**G(iii).Interior view of PSP692 at pH 6 in ligand bound and unbound states**

 (0.781139731,   -0.387079418,   -0.489885181,

     0.623759866,    0.518020213,    0.585299492,

     0.027216038,   -0.762771249,    0.646093786,

     0.000019201,    0.000023842, -145.260864258,

    -0.654262066,   -3.265473843,    3.196613789,

   121.892120361,  168.627700806,  -20.000000000)

ST1H. pH dependence of peptidase activity.

Equal volumes of crude lysates of induced cells expressing PSP692 or PSP464 were plated on 1% gliadin agar plates of pH 4, 6 or 7. Plates were incubated at 37ºC for 6h. The zones of clearance (white arrows) show that both PSP692 and PSP464 have low to no proteolytic activity at pH 7.


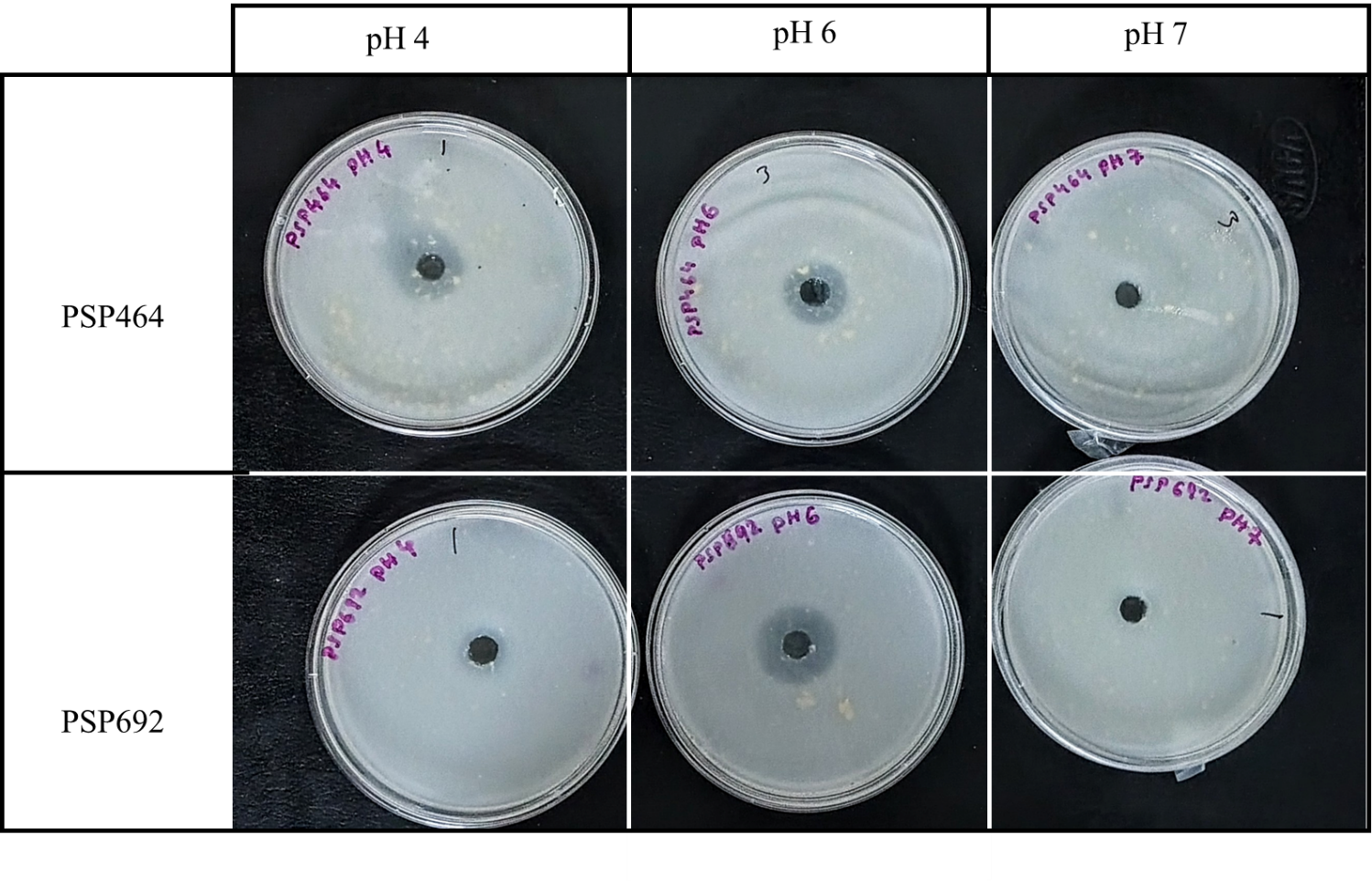

Supplement: Supplemental material [file spectrum.03214-25-s0001.docx]
