# Supplementary material for: Harnessing gut microbiome enzymes: Segatella copri and Stenotrophomonas maltophilia prolyl peptidases degrade gliadin peptides and improve epithelial barrier function in a celiac disease model
Source: Microbiol Spectr. 2026 May 29;14(7):e03214-25. doi: 10.1128/spectrum.03214-25 (PMC13339814; doi:10.1128/spectrum.03214-25)
Supplement: Reviewer comments [file reviewer-comments.pdf]

AMERICAN SOCIETY FOR MICROBIOLOGY | Microbiology Spectrum

Harnessing Gut Microbiome Enzymes: *Segatella copri* and *Stenotrophomonas maltophilia* prolyl peptidases degrade gliadin peptides and improve epithelial barrier function in a Celiac Disease model

Bhagyashree Karmarkar and Dhiraj Dhotre

Corresponding Author(s): Dhiraj Dhotre, National Centre for Cell Science

Review Timeline:

Submission Date:	October 7, 2025
Editorial Decision:	January 25, 2026
Revision Received:	March 22, 2026
Accepted:	April 13, 2026

Editor: Zhe LYU

Reviewer(s): Disclosure of reviewer identity is with reference to reviewer comments included in decision letter(s). The following individuals involved in review of your submission have agreed to reveal their identity: Judy J Brown (Reviewer #1); Muhammad IMRAN (Reviewer #2)

Transaction Report:

(Note: With the exception of the correction of typographical or spelling errors that could be a source of ambiguity, letters and reports are not edited. The original formatting of letters and referee reports may not be reflected in this compilation.)

DOI: https://doi.org/10.1128/spectrum.03214-25

Re: Spectrum03214-25 (**Harnessing Gut Microbiome Enzymes: *Segatella copri* and *Stenotrophomonas maltophilia* prolyl peptidases mitigate gluten immunogenicity in a Celiac Disease model**)

Dear Ms. Bhagyashree Karmarkar:

Thank you for the privilege of reviewing your work. Below you will find my comments, instructions from the Spectrum editorial office, and the reviewer comments.

I apologize for the long delays. The reviewers find your work significant, but they also have reservations due to several concerns. Please address these concerns accordingly. Given the delays, I may not send it out for a second round of review if the revision is done with high rigor. Please make sure 1) a point-to-point rebuttal is provided, 2) both a clean and tracked versions are submitted, 3) clearly state the specific contribution and conclusion of any new experiments done, 4) for the experiments that can not be done, please provide specific reasons, 5) discuss the limitations of the study if concerns from the reviewers can not be addressed fully, and 6) update the data availability statement accordingly if new data is generated.

Please return the manuscript within 60 days; if you cannot complete the modification within this time period, please contact me. If you do not wish to modify the manuscript and prefer to submit it to another journal, notify me immediately so that the manuscript may be formally withdrawn from consideration by Spectrum.

Revision Guidelines
To submit your modified manuscript, log into the submission site at https://spectrum.msubmit.net/cgi-bin/main.plex. Go to Author Tasks and click the appropriate manuscript title to begin. The information you entered when you first submitted the paper will be displayed; update this as necessary. Note the following requirements:

• Upload point-by-point responses to the issues raised by the reviewers in a file named "Response to Reviewers," NOT in your cover letter.
• Upload a compare copy of the manuscript (without figures) as a "Marked-Up Manuscript" file.
• Upload a clean .DOC/.DOCX version of the revised manuscript and remove the previous version.
• Each figure must be uploaded as a separate, editable, high-resolution file (TIFF or EPS preferred), and any multipanel figures must be assembled into one file.
• Any supplemental material intended for posting by ASM should be uploaded with their legends separate from the main manuscript. You can combine all supplemental material into one file (preferred) or split it into a maximum of 10 files with all associated legends included.

For complete guidelines on revision requirements, see our Submission and Review Process webpage. Submission of a paper that does not conform to guidelines may delay acceptance of your manuscript.

Data availability: ASM policy requires that data be available to the public upon online posting of the article, so please verify all links to sequence records, if present, and make sure that each number retrieves the full record of the data. If a new accession number is not linked or a link is broken, provide Spectrum production staff with the correct URL for the record. If the accession numbers for new data are not publicly accessible before the expected online posting of the article, publication may be delayed; please contact production staff (Spectrum@asmusa.org) immediately with the expected release date.

Publication Fees: For information on publication fees and which article types are subject to charges, visit our website. If your manuscript is accepted for publication and any fees apply, you will be contacted separately about payment during the production process; please follow the instructions in that e-mail. Arrangements for payment must be made before your article is published.

ASM Membership: Corresponding authors may join or renew ASM membership to obtain discounts on publication fees. Need to upgrade your membership level? Please contact Customer Service at Service@asmusa.org.

The ASM Journals program strives for constant improvement in our submission and publication process. Please tell us how we can improve your experience by taking this quick Author Survey.

Thank you for submitting your paper to Spectrum.

Sincerely,
Zhe LYU
Editor
Microbiology Spectrum

Reviewer #1 (Comments for the Author):

The authors presented a logical work flow, along with several functionality studies to identify unique microbial peptidases capable of degrading the inflammatory protein, gluten. PSP692 and PSP464 were previously unannotated, which further illustrates the importance of this multi-facetted approach. This reviewer did suggest two major revisions regarding the results of Figure 4 and a control to be added to experiments in Figure 5. Overall, this work shows a rigorous, novel approach to identifying unique and functionally relevant peptidases for the treatment of celiac disease.

Reviewer #2 (Comments for the Author):

This manuscript presents a structure guided metagenomic mining strategy to identify gut microbiome derived prolyl peptidases that can degrade immunogenic gliadin peptides relevant to celiac disease. Two enzymes PSP692 and PSP464 are identified through an integrated computational pipeline and validated through recombinant expression and epithelial cell assays. The study is ambitious and conceptually strong. The integration of metagenomic assembly structure prediction docking molecular dynamics and functional validation is a clear strength. The idea of using complementary enzymes that target different gliadin epitopes is particularly interesting and represents a meaningful improvement over single enzyme approach. However, there are several missing experimental controls and mechanistic validations, and some conclusions go beyond the direct evidence provided. Addressing these issues will significantly improve the rigor and credibility of the study.

1. A major limitation is the lack of direct biochemical confirmation of gliadin cleavage. The selection of enzymes and proposed cleavage mechanisms rely heavily on docking and molecular dynamics predictions. While the epithelial assays suggest functional effects there is no direct evidence showing where and how these enzymes cleave the native gliadin peptides. Without peptide fragmentation analysis it is not possible to confirm that the known immunogenic epitopes are fully destroyed rather than only partially processed. Performing LC MS or HPLC based mapping of digestion products for the 33 mer and 11 mer peptides would greatly strengthen the mechanistic foundation of the work.
2. The kinetic characterization is performed using small chromogenic substrates which do not represent the structural complexity of real gliadin peptides. Therefore, the reported catalytic efficiencies cannot be confidently extrapolated to physiological gliadin degradation. Including kinetic assays using synthetic 33 mer and 11 mer peptides quantified through chromatographic methods would provide more biologically relevant insight.
3. The manuscript frequently states that the enzymes mitigate gluten immunogenicity. However, the only functional readouts used are epithelial stress responses in Caco two cells. No adaptive immune assays such as HLA DQ peptide presentation or T cell activation experiments are included. Therefore, the current data support reduced epithelial damage rather than direct reduction of immunogenicity. The language should be adjusted accordingly to avoid overinterpretation.
4. In the cell-based assays untreated peptide treated and enzyme digested peptide conditions are compared. However, enzyme only controls are not included. These controls are important to rule out any direct effects of recombinant enzymes on epithelial cells independent of peptide digestion. Including enzyme only treatments in IL six TEER transcript and confocal experiments is recommended.
5. The concentrations of peptide and enzyme used in vitro are not justified in relation to physiological intestinal exposure. Demonstrating dose response behavior would strengthen translational relevance and provide a clearer basis for therapeutic claims.
6. The translational discussion would also benefit from addressing safety context. Stenotrophomonas maltophilia is known as an opportunistic pathogen in clinical settings. Although the study uses recombinantly expressed enzymes rather than live bacteria it would be appropriate to briefly clarify this to avoid safety concerns.
7. Finally, only two publicly available celiac metagenomic datasets are used. While this is acknowledged broader claims regarding microbiome protective functions should be presented cautiously given the limited dataset diversity.

Overall, the manuscript presents a creative and technically impressive discovery pipeline with promising in vitro functional validation. With direct biochemical confirmation of gliadin cleavage inclusion of missing experimental controls and careful moderation of immunological claims the study has strong potential for publication in a high-quality journal. I feel this paper inappropriate to be publish in current form.

Harnessing Gut Microbiome Enzymes: *Segatella copri* and *Stenotrophomonas maltophilia* prolyl peptidase mitigate gluten immunogenicity in a Celiac Disease model

Bhagyashree Karmarkar, Dhiraj Dhotre

The article, “Harnessing Gut Microbiome Enzymes: *Segatella copri* and *Stenotrophomonas maltophilia* prolyl peptidase mitigate gluten immunogenicity in a Celiac Disease (CeD) model” describes a multi-facetted, discovery-based method to identify previously unannotated peptidases from microbial species. The enzymes, produced by microbial species specific to the microbiome of non-Celiac disease patients, were predicted to have glutenase activity. Previous work has shown that adjuvant enzyme therapy with glutenases has the potential to mitigate the toxicity of gluten, the inflammatory peptide required for the development of CeD. Several studies have identified microbial peptidases capable of digesting immunodominant peptides, but the process can be labor intensive and potentially biases some targets. The authors sought to develop a strategy to stream-line discovery of peptidases, using metagenomic data from human cohorts of patients with and without CeD, optimized for taxonomic and functional fidelity. Uniquely, this strategy identified peptidases genes that were structurally and functionally convergent but may have been missed by alignment-based strategies or due to low abundance. This screening approach led to the discover of two novel glutenases, PSP692 from *Segatella copri* and PSP464 from *Stenotrophomonas maltophilia*. Both enzymes were validated for gluten-specific enzymatic activity using physiologically relevant conditions and evaluated for ability to mitigate inflammatory conditions in cell culture. The degradation of the inflammatory gliadin epitopes with either PSP692 or PSP464 restored the expression of tight junction proteins (ZO-1 and occuldin), reduced the section of IL-6, and improved barrier function in CaCo-2 cells. Although they do share common motifs, PSP692 and PSP464 do not cluster with other known proline-specific proteases in sequence, which highlights how this method is able to identify unique but functionally related proteins from clinically relevant microbial

populations. The authors describe how this approach could be applied to ever-growing datasets of gluten epitopes and microbiome cohort studies to identify novel proteases with improved stability and functionality. Such microbial enzymes could be used for combination adjuvant therapy or introduced into recombinant duodenal bacteria. This study adds another data point to the discussion of what role the microbiota can play in mitigating the deleterious effects of CeD.

Major Revisions

1. Figure 4 does not show the relationship described in the Results section or in the legend. According to Figure 4A and the corresponding key, PSP464 (E2) restores ZO-1 expression for the 33 mer and PSP692 (E1) restores ZO-1 expression of 5ksb/11mer. The authors should address this discrepancy.

2. The quantification of ZO-1 expression at CaCo-2 cell surface should be repeated with an untreated negative control void of both 33-mer and enzyme. Such a control would be consistent with experiments for ZO-1 transcripts in Figure 4. Images of staining with ZO-1 and DAPI should be added to Figure 5 along with graphical quantification. This would allow for visualization of the typical pattern of ZO-1 on CaCo-2 cells.

Minor Revisions

1. Methods section 2.11 should include the method used to quantify ZO-1 expression for Figure 5.

2. Results Section 3.1 should include discussion of results from Figure 2C-I.

3. Figure 2C and D should be edited so that they are easier to see, the font size is very small.

4. Results Section 3.2 should include discussion of the results from Figure 3A.

5. Results Section 3.2 “Expression of ZO-1 and Occludin” should be edited to include more information about why these two transcripts were selected. This reviewer suggests that the following statement be placed at the beginning of the paragraph in line 316 to give the reader more introduction to this section:

   “ZO-1 is a critical component of intestinal tight junctions, functioning as a platform for the attachment of occludin and claudins at intercellular site and for adherence of actin cytoskeleton on cytoplasmic side; overall this assembly plays a pivotal role in the regulation of barrier function.”

6. Figure 4 and legend could be edited for consistency and clarity:
   a. The legend states that Figure 4C shows secretion of IL-6 but the Figure has IL-6 section labelled as 4B.
   b. Figure 4D does not exist in the Figure but is included in the Figure legend.
   c. The right panels of Figure A confused this reader with the label “5ksb.” This peptide name was only used in the methods section but not in the Results or Figure legend. Changing the x axis labels to 11-mer (orange), E1-11 (green), and E2-11 (salmon) would be consistent with the Sample key, left side of Figure 4A, and the Figure legend.
   d. The Sample key could be boxed or separated if it is used for the other Figures. At present, the Sample key looks like it only refers to Figure 4C.

7. Line 31-32 should be reworded for clarity, “We have outlined and validated a method to identify enzymes with potential from metagenomic data that will also be validated experimentally.”

8. Line 272-274 states, “Phylogenetic tree was constructed in MEGA1237 to compare our recombinant peptidases with 272 bacterial peptidases that are validated to have gluten degrading activity (**Figure 1A**) and with 273 various peptidases predicted from metagenomic data and annotated in MEROPS(**Figure 1B**).” These lines should be edited as follows:

   “Phylogenetic tree was constructed in MEGA1237 to compare our recombinant peptidases with 272 bacterial peptidases that are validated to have gluten degrading activity (**Figure 2A**) and with 273 various peptidases predicted from metagenomic data and annotated in MEROP**S (Figure 2B**).

9. Line 287-292 contains a repeated sentence, “We further determined from phylogenetic analysis of protein sequences of PSP464 and PSP692 that our query peptidases cluster separately from previously validated gluten degrading enzymes.”

10. Line 298 should be reworded, “which also underlies the ability of these enzymes to degrade immunogenic gliadin.”

11. Line 317 should say “**undigested**”

12. Line 345 states that, “PSP464 also demonstrated significant efficacy.” According to the quantification data in Figure 4C, the green bar labelled “E1-33” was not significant.

13. Line 434-436 should be edited as follows:

   “IL-6 secretion was reduced to near-baseline levels (Figure 4**B**), and transcript levels of tight junction marker**s** were restored significantly (Figure 4A~~-B~~)

14. Line 457 states, “Our data suggest that combining enzymes with complementary cleavage preferences can enable a more thorough and effective degradation of

gluten peptides in the gastrointestinal tract." The authors did not perform any studies in which enzymes were used in combination.

15. The Discussion would benefit from text stating how this new methodology for identifying microbial proteins could be expanded outside of CeD research.

16. This reviewer is curious about whether there were overlapping extracellular peptidase sequences identified by the two methods (metagenomic and database approach) and which approach led to the discovery of the two novel proteins.

17. This reviewer is curious whether phage therapy could introduce such beneficial enzymes into already existing duodenal bacteria.

Reviewer comments

1. We thank the reviewer for highlighting the importance of direct biochemical confirmation of gliadin cleavage. To address this concern, we performed HRMS-based mapping of synthetic 33-mer and 11-mer peptides incubated with the recombinant enzymes. Time-course chromatographic analysis revealed progressive disappearance of the intact peptide peak accompanied by the appearance of multiple lower-retention fragments, indicating direct enzymatic cleavage. The intact 33-mer was no longer detectable after 30 minutes under the tested conditions (Figure 4A), similarly the intact 11-mer was also no longer detectable in digested samples (Figure 4B). While LC-MS-based fragmentation analysis would provide site-specific resolution, the present chromatographic data directly demonstrate enzymatic degradation of the gliadin peptides and substantiate the mechanistic conclusions.
2. We thank the reviewer for this important comment. We agree that chromogenic substrates such as Z-PPF-pNA and Z-GP-pNA do not fully capture the structural complexity of native gliadin peptides. These substrates were used primarily for initial biochemical characterization of enzymatic activity, which is a commonly adopted approach for prolyl peptidases.

   To address the physiological relevance of gliadin degradation, we complemented these assays with direct digestion experiments using native gliadin peptides. HRMS analysis demonstrated time-dependent degradation of the 11-mer and 33-mer gliadin peptides, with a decrease in the parent ion intensity and the appearance of fragment ions (m/z ~872 and ~998 for the 11-mer, and m/z ~697 and ~725 for the 33-mer). These results provide direct evidence that PSP464 and PSP692 are capable of degrading gliadin peptides beyond the simplified chromogenic substrates.

   We agree that detailed kinetic analysis using intact gliadin peptides quantified through chromatographic approaches would provide additional insight. However, such measurements are technically challenging due to the heterogeneous cleavage patterns of these peptides, the appearance of multiple intermediate fragments at single time points and the absence of extensive LC-MS data for fragment assignment and were beyond the scope of the present study. We have clarified this limitation in the revised manuscript and moderated the interpretation of catalytic efficiency accordingly.

3. We agree that T-cell proliferation assays would provide direct evidence of reduced immunogenicity. However, these assays require specialized immunological models beyond the scope of the present study. To address this concern, we have moderated statements regarding immunogenicity throughout the manuscript and now focus on enzymatic degradation of gliadin peptides and improvement of epithelial barrier markers.
4. We thank the reviewer for this suggestion. The gliadin digestion reactions were performed using purified recombinant enzymes. These enzymes exhibit activity only under acidic conditions and are inactive at neutral pH (~7) (supplemental figure ST1H), which corresponds to the pH of the cell culture medium used in the immunological assays. Therefore, enzymatic activity would not be expected to occur during cell exposure. Hence the observed cellular responses reflect differences in gliadin peptide composition generated during the digestion step rather than direct effects of the enzyme preparation.
5. Based on clinically employed gluten re-challenge protocols (3–7.5 g/day) (Leffler et al., 2012; Catassi et al., 2007), the α-gliadin fraction of wheat gluten (~28–33%) (Wieser, 2007; Shewry & Halford, 2002), and reported post-prandial gastric volumes of approximately 400–600 mL (Marciani et al., 2001; Hoad et al., 2004), the effective luminal concentration of immunogenic gliadin peptides can be estimated to fall within the sub- to low-millimolar range. Considering the molecular weight of the immunodominant 33-mer α-gliadin peptide (~3.9 kDa) (Shan et al., 2002; Shan et al., 2005), this corresponds to an approximate concentration range of ~0.36–1.27 mM under gastric conditions. Enzyme–substrate molar ratios of 1:500 therefore correspond to low-micromolar enzyme concentrations (~1.5–3 µM assuming a mid-range substrate concentration of ~0.75 mM), consistent with catalytic proteolysis of gliadin peptides by exogenous glutenases (Shan et al., 2004; Bethune & Khosla, 2008), and with enzyme concentration frameworks employed in physiologically relevant in vitro digestion models such as the INFOGEST consensus protocol, where enzyme activities are standardized per unit volume of digesta to approximate in vivo gastric conditions (Minekus et al., 2014). Notably, Phase I clinical evaluation of the engineered glutenase TAK-062 (Kuma062) has employed enzyme doses in the range of 300–900 mg (Pultz et al., 2021; Takeda clinical reports), corresponding to substantially higher enzyme:substrate molar ratios (~1:10 to 1:40), thereby providing an independent clinical

benchmark indicating that enzyme concentrations in the range of 1:500 remain well within a physiologically and therapeutically relevant regime for effective gluten detoxification.

6. The clarification regarding the use of recombinantly expressed enzyme from Stenotrophomonas maltophilia, by itself not associated with the organisms opportunistic pathogenicity has been included in Discussion section in lines 505-509.

Major revisions

1. The legend for former Figure 4 now Figure 5 has been corrected to represent the experimental results. Throughout the manuscript PSP692 is E2 which degrades 33-mer peptide while PSP464 is E1 which degrades 11-mer peptide.

2. Confocal imaging of control CaCo-2 cells void of both enzyme and peptide has been added. Cell culture (seeding number, growth duration) and imaging (laser power, focal depth) was performed under conditions identical to those for peptide only and digested peptide conditions as reported in Methods 2.12.

Minor revisions

1. Methods section 2.11 has been amended to include that the software used to quantify ZO-1 fluorescence is ImageJ, appropriate citation has been added.
2. We have expanded section 3.1 to include detailed discussion of results represented in Figure 2C-I.
3. Figure 2C and 2D have been edited for clarity of labels within the image.
4. Result displayed in figure 3A have been added to results section 3.2
5. We thank the reviewer for their suggestion and have included the suggested statement at line 316.
6. We thank the reviewers for carefully noting these inconsistencies. The figure numbering and legend have been corrected to ensure alignment between panels and descriptions. The mislabeled IL-6 panel has been amended, the erroneous reference to Figure 4D has been removed, and the x-axis labels in Figure 4A have been revised to “11-mer,” “E1-11,” and “E2-33” for consistency with the sample key and legend. Additionally, the Sample key has been reformatted and clearly separated to avoid ambiguity and to indicate its applicability across the relevant panels.

7. Line 31-32 has been reworded for added clarity to "We present a systematic approach for identification of potential gluten degrading enzymes from metagenomes and databases, coupled with experimental validation."
8. Lines 272-274 have been edited to cite the correct Figure.
9. Sentence repeated in lines 287-292 has been deleted.
10. Line 298 has been reworded to 'Thus, despite belonging to the S8/S9 class of enzymes, proline-specific peptidases exhibit subclass-dependent substrate specificity, which underlies the differences in their ability to degrade gliadin."
11. Typographic error on line 317 has been rectified.
12. Line 345 has been removed to reflect the data represented.
13. Lines 434-436, now lines 487-488 has been corrected to cite the correct figure panel.
14. Line 457, now line 511 has been corrected to "Enzymes with complementary cleavage preferences may act synergistically to enable more thorough and effective degradation of gluten peptides in the gastrointestinal tract."
15. We have included a small section within Discussion on the applicability of methodology described in our manuscript to other food antigen induced conditions.
16. We did not observe overlapping extracellular peptidase sequences between the metagenomic screening and the database-driven approach. This is primarily because the database mining strategy applied an additional filtering criterion: the selection of peptidases from aerobic organisms that were amenable to laboratory cultivation. This was applied to enable genomic DNA isolation and downstream cloning and expression. In contrast, the metagenomic approach was culture-independent and therefore captured a distinct pool of sequences, including those from uncultivable or anaerobic organisms.
17. We thank the reviewer for this insightful question. Phage-based delivery of therapeutic proteins is an emerging and intriguing strategy, and there are indeed reports demonstrating the use of bacteriophages for in situ expression or display of heterologous proteins in the gut.

    However, the applicability of such an approach in the context of celiac disease raises several unresolved challenges. First, the infectivity and persistence of a given phage depends strongly on the presence and abundance of susceptible bacterial hosts. The duodenal microbiota in celiac disease is known to be dysbiotic, and it remains unclear whether

canonical T4/Jug strains are sufficiently represented or accessible in this environment to enable reliable delivery.

Second, phage-mediated expression introduces additional layers of complexity related to protein folding, processing, and potential post-translational or assembly-associated modifications during phage production. Such modifications could alter antigenicity or immunogenicity of the expressed enzyme, which is a particularly important consideration in an immune-mediated disorder such as celiac disease.

For these reasons, while phage-based delivery is conceptually attractive, its use for targeted enzyme delivery in the celiac gut would require substantial additional validation of host range, expression fidelity, and immunological safety. In the present work, we therefore focus on direct functional characterization of the enzyme itself, which represents a necessary first step irrespective of the eventual delivery platform.

Re: Spectrum03214-25R1 (**Harnessing Gut Microbiome Enzymes: *Segatella copri* and *Stenotrophomonas maltophilia* prolyl peptidases degrade gliadin peptides and improve epithelial barrier function in a Celiac Disease model**)

Dear Mr. Dhiraj Prakash Dhotre:

Your manuscript has been accepted, and I am forwarding it to the ASM production staff for publication. Your paper will first be checked to make sure all elements meet the technical requirements. ASM staff will contact you if anything needs to be revised before copyediting and production can begin. Otherwise, you will be notified when your proofs are ready to be viewed.

Data Availability: ASM policy requires that data be available to the public upon online posting of the article, so please verify all links to sequence records, if present, and make sure that each number retrieves the full record of the data. If a new accession number is not linked or a link is broken, provide production staff with the correct URL for the record. If the accession numbers for new data are not publicly accessible before the expected online posting of the article, publication may be delayed; please contact ASM production staff immediately with the expected release date.

Publication Fees: For information on publication fees and which article types have charges, please visit our website. We have partnered with Copyright Clearance Center (CCC) to collect author charges. If fees apply to your paper, you will receive a message from no-reply@copyright.com with further instructions. For questions related to paying charges through RightsLink, please contact CCC at ASM_Support@copyright.com or toll free at +1-877-622-5543. CCC makes every attempt to respond to all emails within 24 hours.

ASM Membership: Corresponding authors may join or renew ASM membership to obtain discounts on publication fees. Need to upgrade your membership level? Please contact Customer Service at Service@asmusa.org.

Embargo Policy: A press release may be issued as soon as the manuscript is posted on the Spectrum Latest Articles webpage. The corresponding author will receive an email with the subject line "ASM Journals Author Services Notification" when the article is available online.

The ASM Journals program strives for constant improvement in our submission and publication process. Please tell us how we can improve your experience by taking this quick Author Survey.

Thank you for submitting your paper to Spectrum.

Sincerely,
Zhe LYU
Editor
Microbiology Spectrum

Reviewer #1 (Comments for the Author):

The authors have sufficiently satisfied all of the concerns of this reviewer.

Reviewer #2 (Comments for the Author):

NA
